# Learning shapes the development of migratory behavior

**DOI:** 10.1073/pnas.2306389121

**Published:** 2024-03-04

**Authors:** Ellen O. Aikens, Elham Nourani, Wolfgang Fiedler, Martin Wikelski, Andrea Flack

**Affiliations:** ^a^School of Computing, University of Wyoming, Laramie, WY 82071; ^b^Haub School of Environment and Natural Resources, University of Wyoming, Laramie, WY 82072; ^c^Collective Migration Group, Max Planck Institute of Animal Behavior, Radolfzell 78315, Germany; ^d^Centre for the Advanced Study of Collective Behaviour, University of Konstanz, Konstanz 78468, Germany; ^e^Department of Migration, Max Planck Institute of Animal Behavior, Radolfzell 78315, Germany; ^f^Department of Biology, University of Konstanz, Konstanz 78457, Germany

**Keywords:** ontogeny of migration, fidelity, timing, routes, energetics

## Abstract

How animals refine migratory behavior over their lifetime (i.e., the ontogeny of migration) is an enduring question with important implications for predicting the adaptive capacity of migrants in a changing world. Yet, our inability to monitor the movements of individuals from early life onward has limited our understanding of the ontogeny of migration. The exploration–refinement hypothesis posits that learning shapes the ontogeny of migration in long-lived species, resulting in greater exploratory behavior early in life followed by more rapid and direct movement during later life. We test the exploration–refinement hypothesis by examining how white storks (*Ciconia ciconia*) balance energy, time, and information as they develop and refine migratory behavior during the first years of life. Here, we show that young birds reduce energy expenditure during flight while also increasing information gain by exploring new places during migration. As the birds age and gain more experience, older individuals stop exploring new places and instead move more quickly and directly, resulting in greater energy expenditure during migratory flight. During spring migration, individuals innovated novel shortcuts during the transition from early life into adulthood, suggesting a reliance on spatial memory acquired through learning. These incremental refinements in migratory behavior provide support for the importance of individual learning within a lifetime in the ontogeny of long-distance migration.

## Significance

Migratory decision-making is often conceptualized as reducing either time or energy expenditure; however, the importance of these currencies may shift throughout life. The exploration–refinement hypothesis suggests that information is an additional currency shaping the lifetime development of migration, predicting that exploration and information gain should be favored early in life. Using a unique early-life tracking dataset, we show that white storks incrementally refine migration timing and routes by innovating novel shortcuts during migration. Storks switch from energy-efficient exploration to rapid and directed movement as they age. Together, these results suggest that learning and early-life exploration play an important role in the ontogeny of migration in a long-lived migratory bird and that information is a critical currency shaping migration.

Complex behaviors often change over an animal’s lifetime. This is especially true when the selective pressures shaping the behavior shift as the animal ages. Studying how behaviors develop is a core foundation of behavioral ecology, referred to as ontogeny (1). The complex and taxonomically widespread behavior of animal migration requires many decisions about when, where, and how to move (2, 3). The outcome of these decisions impacts individual fitness (4), population dynamics (5), community ecology (6), and ecosystem functioning (7). Yet, despite the importance of migratory behavior, how migration is established and refined over an animal’s lifetime—what we refer to as the ontogeny of migration—is largely unknown for most species (8–10).

Decision-making during migration is often conceptualized as a tradeoff between different optimization criteria or “currencies” required for successful migration (11), with energy and time being the two most studied currencies (12, 13). Migratory animals may benefit from reducing the amount of energy expended per distance traveled (14), which may be particularly relevant for flying and swimming migrants that can use uplift, wind, or currents to facilitate long-distance movements with minimal energy (15, 16). Alternatively, many migrants face selective pressures to arrive at their destination early when there is competition for limited resources at migratory destinations (17). For example, in species that require high-quality territories to successfully reproduce, the pressure to arrive early to secure and defend critical resources may result in faster and earlier migrations that are energetically costly (17). Examining the currencies that migrants must balance and how the relative importance of these currencies shifts over a migrant’s lifetime will likely provide important insights into the ontogeny of migration (11).

Although information has largely been overlooked as a currency shaping migratory behavior, gaining information and using it to incrementally refine migration behavior through learning could play an important role in saving both energy and time. The landscapes that animals move through are complex and dynamic, requiring that migrants learn where and when favorable conditions that facilitate movement occur and how to exploit them efficiently (18, 19). For example, several studies comparing juvenile and adult birds indicate that older birds used less energy during flight, presumably because they learned to fly more efficiently as they gain experience (19, 20) and perhaps also because they learned energy-efficient movement paths. Energy-efficient movement paths are likely to be circuitous to avoid areas that are costly to move through (e.g., steep terrain, strong headwinds; refs. 21 and 22). When time is of the essence, however, more direct routes may be favored and there may even be selective pressure to shorten migration (12, 23–25). Moving directly over long distances during migration likely also requires detailed knowledge of the environment, acquired through learning and spatial memory (26–28). For example, only after gaining detailed knowledge of their environment, did fruit bats learn shortcuts that allowed them to move more directly between key locations (29, 30). Thus, detailed knowledge of the landscape may be a prerequisite to moving in a directed and time-minimizing manner during migration (26).

The relative importance of energy, time, and information may shift during the ontogeny of migration. For a long-distance migratory bird, learning to fly efficiently likely occurs early because efficient flight is under significant selective pressure from the onset of life (31). For example, juvenile albatrosses (*Thalassarche chrysostoma*) quickly learn how to efficiently exploit favorable wind conditions to travel large distances (32). In contrast, completing migration earlier to secure breeding territories or nest sites likely becomes important only during the reproductive phase of life. The exploration–refinement hypothesis helps to conceptualize how animals might balance energy, time, and information as they perform multiple migrations over their lifetime (33). Specifically, it predicts that exploratory behavior should be favored in early life to allow migrants to gain critical information about seasonal ranges or migration routes, but as migrants enter into the reproductive phase of life, they are predicted to move more directly and quickly between migratory destinations (10, 25, 33). Importantly, the exploration–refinement hypothesis suggests that the gradual refinement of migration routes and timing within a lifetime provides evidence of the critical role of individual learning in the ontogeny of migration (25, 33). We extend the exploration–refinement hypothesis by predicting that in addition to age-related changes in migration timing and routes, energetic expenditure during migratory flight is likely to change as well. Simultaneously examining how flight energetics, timing, and routes shift from early life onward is key in understanding the role of learning in migration development, while elucidating potential trade-offs migrants face in balancing the relative importance of energy, time, and information during the ontogeny of migration.

Using a unique tracking and biologging dataset that recorded movements and activity levels from fledging onward, we tested the exploration–refinement hypothesis in white storks (*Ciconia ciconia*; (34–38)). From June 2013 to 2020, we deployed solar-powered GSM tags on over 250 juvenile white storks spread across five breeding areas in southern Germany and Austria (*SI Appendix*, Table S1) that use the western flyway (i.e., movements toward Northwestern Africa via the strait of Gibraltar; see ref. 24 for details). This seven-year data collection effort (June 2013–April 2021) resulted in 301 migration events collected from 40 white storks that completed a minimum of two consecutive fall migrations (*SI Appendix*, Table S2). Previous work in this system has established that selective mortality occurring during the first fall migration plays a key role in shaping migration distance and destination (24). Here, we focused on the ontogeny of three key components of migration: timing (i.e., when to move), routes (i.e., where to move), and flight energetics (i.e., how to move). White storks face pressure to reduce both time and energy expenditure during migration (39), and their relatively long lives [average life expectancy = 8 to 9 y (40)] provide ample opportunity to refine migration through learning. Like many other birds that are adapted for soaring flight, storks use rising columns of air to gain altitude and cover large distances with minimal energy (41, 42). Thus, energy-efficient flight is likely to be essential during long-distance migrations. Yet, the rapid growth of many stork populations, including the populations in this study, results in competition for limited nesting sites that require significant investments to build, maintain, and defend (43, 44), increasing pressure to complete spring migration early. We tested and extended the exploration–refinement hypothesis by examining how migration timing, routes, and flight energetics shifted as white storks aged and gained experience. We estimated information gain and learning from metrics of the route that provide evidence of exploration (i.e., low fidelity to previous routes) and exploitation [i.e., moving directly between migration destinations and innovating shortcuts during migration (26)].

## Results

### Storks Migrate Faster as they Age.

**Fig. 1.**
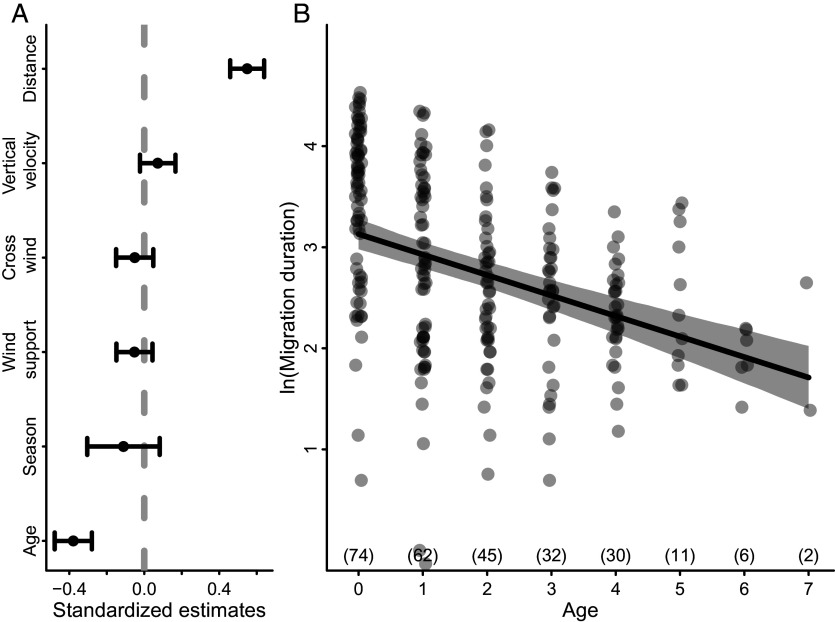
The influence of age on migration duration for white storks (*Ciconia ciconia*) that were born in southern Germany and tracked continuously from early life onward. (*A*) The standardized coefficient estimates for fixed effects of the model. (*B*) The fitted relationship between the natural log of migration duration in days and age in years. Numbers in parentheses above the *x* axis in *B* show the sample size for each age class (including autumn and spring events). In *B*, the solid black line shows the fitted relationship, and the gray polygon indicates the 95% CI estimated by semiparametric bootstrapping (n = 1,000 simulations). n = 262 migration events (spring and fall migrations) from 40 individuals.

To determine the importance of time as a currency shaping the ontogeny of migration, we examined the effect of age on migration duration, while controlling for the potentially confounding effects of migration distance, wind support, crosswinds, uplift strength (estimated using vertical velocity, ref. 31), and season (i.e., fall or spring migration). As individuals aged, migration duration decreased (Fig. 1 and *SI Appendix*, Table S3). Distance was the only other variable included in the model that had a significant effect on migration duration (Fig. 1*A* and *SI Appendix*, Table S3). In the fall, younger birds started migration earlier and ended migration later than older birds (*SI Appendix*, Fig. S1 and Tables S4 and S5). In spring, younger birds started and ended spring migration later than older birds (*SI Appendix*, Fig. S1 and Tables S6 and S7). We found no evidence that an increased ability of older individuals to compensate for adverse environmental conditions during flight (e.g., drift from crosswinds) influenced migration duration (*SI Appendix*, Fig. S2).

In addition to examining the ontogeny of migration timing and duration, we also explored ontogenetic choices about when to fly during migration in relation to environmental conditions. First, we compared mean environmental conditions (i.e., wind support, crosswinds, and vertical velocity) during periods of flight and nonflight across spring and fall migrations (*SI Appendix*, Fig. S3). Across all age classes, more favorable environmental conditions (i.e., greater wind support, minimal crosswinds, and stronger thermal uplift) were experienced during flight in comparison to nonflight periods for wind support during spring migration (*SI Appendix*, Fig. S3*B*), crosswinds during both fall and spring migration (*SI Appendix*, Fig. S3 *C* and *D*) and uplift strength during fall migration (*SI Appendix*, Fig. S3*E*). Furthermore, we used the difference in environmental conditions experienced during flight and nonflight periods for each migration event to examine whether older animals improved choices about when to fly during migration based on environmental conditions that should enhance flight performance. This analysis revealed no age-specific changes in environmental conditions between flight and nonflight days during fall migration (*SI Appendix*, Fig. S4 and Tables S8–S10). During spring migration, however, older birds experienced more favorable wind support and lower crosswinds but poorer uplift strength during migratory flight in comparison to nonflight periods (*SI Appendix*, Fig. S4 and Tables S8–S10).

### Energy Expenditure during Migration Increases with Age.

**Fig. 2.**
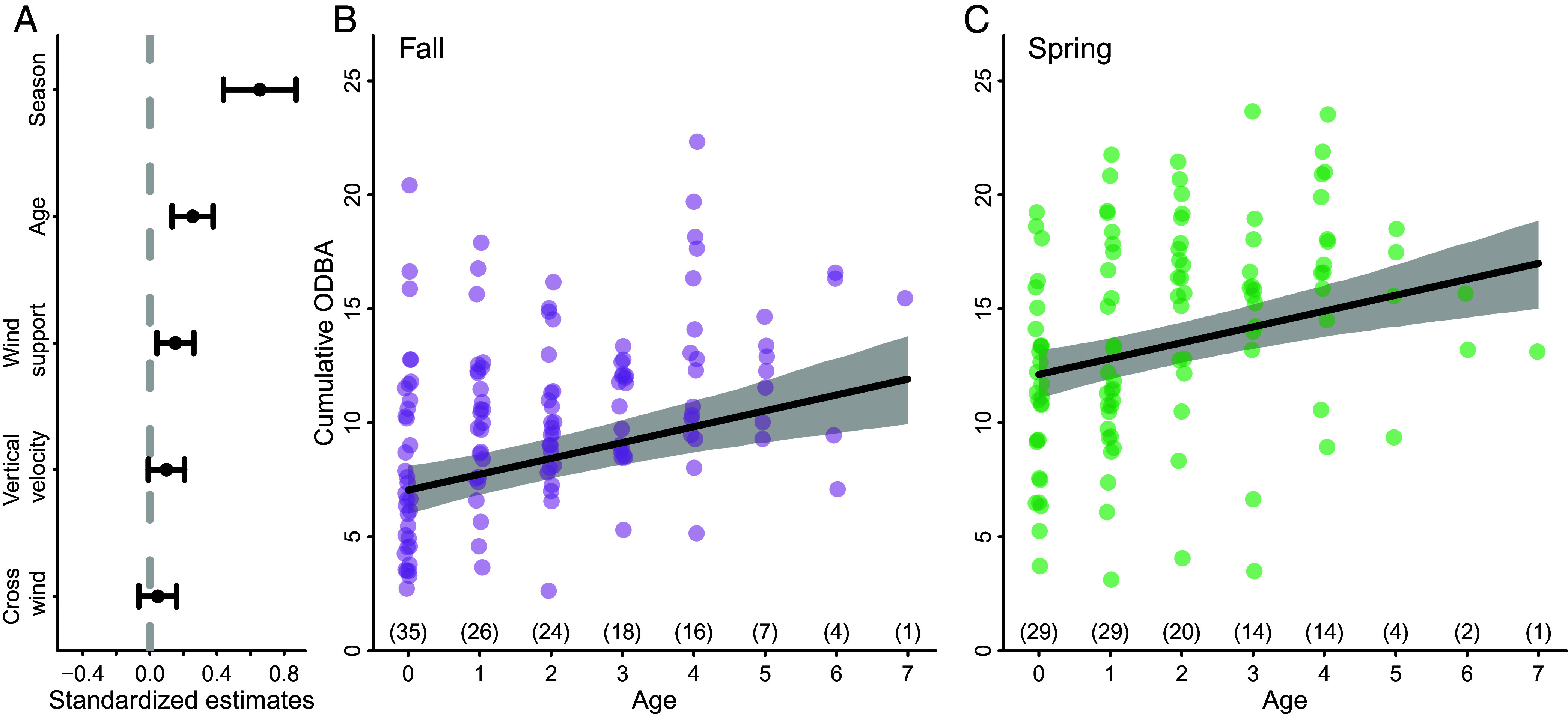
The influence of age on cumulative energy expended during migratory flight by white storks (*Ciconia ciconia*) tagged as juveniles in southern Germany. (*A*) Standardized coefficient estimates for the fixed effects of the linear mixed-effects model built to examine the effect of age on energy expenditure, while controlling for the potentially confounding effects of wind support, crosswinds, vertical velocity (a proxy for uplift strength), and season. Energy expenditure increased with age and was lower during fall migration (purple; *B*) in comparison to spring migration (green; *C*). Energy expenditure is calculated as the cumulative Overall Dynamic Body Acceleration (ODBA) during migratory flight. Numbers in parentheses above the *x* axis in *B* and *C* show the sample size for each age class. The solid black line in *B* and *C* shows the fitted relationship, and the gray polygon indicates the 95% CI estimated by semiparametric bootstrapping (n = 1,000 simulations). n = 244 migration events (spring and fall migrations) from 40 individuals.

To investigate the importance of energy efficiency, we examined the effect of age on energy expenditure during migratory flight, while controlling for season and environmental conditions (i.e., wind support, crosswinds, and uplift). We estimated total energy expenditure during migratory flight using accelerometry (ACC) data that was recorded on a subset of the tagged storks (82.9% of animal years). From ACC recordings, we calculated the Overall Dynamic Body Acceleration (ODBA), which approximates activity and has been correlated with energy expenditure and wingbeat frequency in birds (45, 46). Cumulative ODBA during migratory flight increased with age and was higher during spring migration in comparison to fall migration (*SI Appendix*, Table S11 and Fig. 2). This pattern was similar when ODBA was calculated as energy expenditure per distance traveled during migratory flight (*SI Appendix*, Fig. S5) and when migration duration was included in the model of cumulative ODBA (*SI Appendix*, Fig. S6). We found no evidence that the change in cumulative ODBA with age was impacted by an increased ability of older individuals to compensate for crosswinds (*SI Appendix*, Fig. S2).

### Storks Shift from Exploration to Exploitation.

**Fig. 3.**
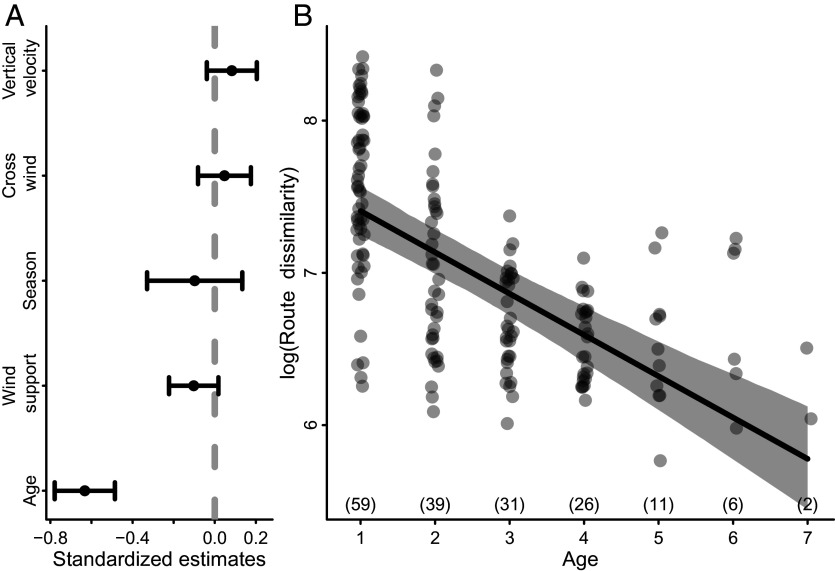
The influence of age on route dissimilarity of white storks tracked from fledging onward. (*A*) Standardized coefficient estimates of the fixed effects of a model examining the impact of age on route fidelity, while controlling for the potentially confounding effects of season and environmental conditions (i.e., wind support, crosswinds, and uplift strength estimated as vertical velocity). (*B*) As individuals aged, route dissimilarity decreased, suggesting that birds are exploratory during early life but become more faithful to their migration route as they age. Route dissimilarity is measured as the log of DTW, where smaller values indicate greater route fidelity. The numbers in parentheses above the *x* axis in *B* indicate the sample size for each age class (including autumn and spring events). In *B*, the solid black line shows the fitted relationship, and the gray polygon indicates the 95% CI estimated by semiparametric bootstrapping (n = 1,000 simulations). n = 174 observations from 39 individuals.

To examine how information shaped migration development, we estimated exploration and exploitation metrics at various scales of migration. Exploration would indicate information gathering, while exploitation would indicate the use of existing information to make more direct movements (26). To quantify exploration, we examined the degree of fidelity individuals exhibited to previous migration routes. Specifically, we used Dynamic Time Warping (DTW), which is a trajectory similarity metric (47). Larger DTW values represent greater route dissimilarity and are indicative of greater exploration during subsequent migrations. To examine the impact of age on route fidelity, we built a model that accounted for season and environmental variability (i.e., wind support, crosswinds, and thermal uplift). As animals aged, they became more faithful to their previous migration route, as indicated by a decrease in route dissimilarity (Fig. 3 and *SI Appendix*, Table S12). Thus, younger individuals were more exploratory, but routes became less flexible as individuals aged. By their second migration, individual routes were consistently more similar to their own previous routes than to all other routes (*SI Appendix*, Fig. S7), excluding the possibility that individuals were following others or converging on an optimal route used by the majority of individuals. We found that changes in route fidelity with age could be explained, in part, by older birds being better able to compensate for drift, as indicated by a weakly significant interaction between age and crosswinds (*SI Appendix*, Figs. S2 and S8).

**Fig. 4.**
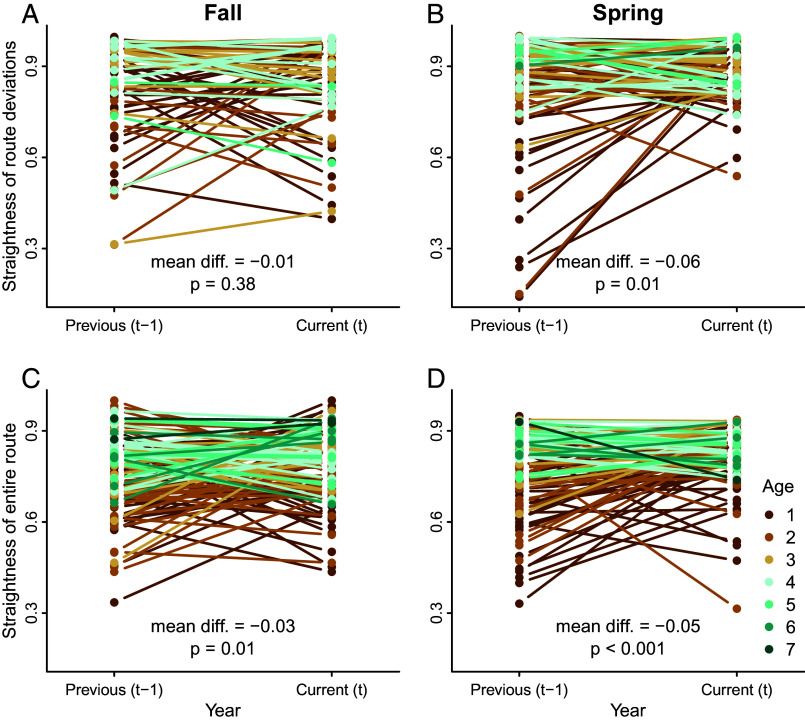
A comparison of trajectory straightness for route deviations (i.e., when individuals deviated from segments of the previous year’s route; *A* and *B*) and migration routes (*C* and *D*) derived from tracking data of white storks tagged as juveniles in southern Germany. Trajectories were compared across fall (*A* and *C*) and spring (*B* and *D*) migrations. A straightness value of one indicates the most direct route (i.e., equivalent to the beeline between start and end locations). Trajectory straightness between previous and current years was compared using paired *t*-tests such that negative estimated differences in means (abbreviated mean diff. in *A*–*D*) indicated straighter trajectories in the current rather than previous years.

To directly quantify exploitation, we compared the length and directness of the entire route across subsequent migrations (*SI Appendix*, Tables S13 and S14). We also compared segments of the route where individuals deviated from previous migration (see *SI Appendix*, Fig. S9 for details) to examine whether individuals were able to innovate shortcuts during migration. As individuals aged, their routes became straighter at the entire route level (*SI Appendix*, Fig. S10). Straightness of fine-scale route deviations and the entire route increased when comparing previous and current migrations; however, the effect size was greater during spring migration, and this effect was not significant for fine-scale route deviations in the fall (Fig. 4). The increasing straightness in fine-scale route deviations from the previous year’s routes in the spring suggests that individuals incrementally refined migration routes through shortcuts as they aged and gained more experience (Fig. 4*B*). We examined the effect of age, season, and environment (uplift, wind support, and crosswinds) on migration distance (measured as the great circle distance between the start and end of migration) but found no significant effects (*SI Appendix*, Table S14), indicating that animals did not innovate short-stopping as a result of increased experience or changes in environmental conditions.

## Discussion

We provide support for the exploration–refinement hypothesis using a unique tracking and biologging dataset that recorded detailed information on both migratory movements and energetics from early life onward for storks that survived to complete at least two consecutive fall migrations. The incremental changes in timing, energetics, and routes suggest that individual learning and information gathering shape the ontogeny of migration in a long-lived bird. Although previous work has compared route characteristics and timing across juveniles and known-age individuals (e.g., refs. 8, 25, and 33), we provide the strongest evidence to date of early-life exploration shaping migration later in life by quantifying characteristics of migration continuously through development. Furthermore, our addition of accelerometry data to quantify flight energetics adds a more nuanced view of the refinement of migratory behavior, revealing that storks switch from an energy-saving to a time-saving strategy as they age (Figs. 1 and 2). Migratory flights with low energy expenditure allow young individuals to explore unfamiliar places, gaining new information about the landscape (Figs. 2 and 3). For young storks, the first migration is an especially risky period and early-life mortality is known to impact population-level shifts in migration distance and destination (24). Thus, adopting an energy-efficient strategy might be necessary to successfully complete migration and gather information in early life. As animals gain knowledge about their migratory environment, they appear to switch to a more energetically costly but rapid movement strategy, which likely enhances their ability to compete for breeding territories and increase reproductive success. Together, these findings highlight that migration is a complex and dynamic behavior that is shaped by learning.

Individual storks incrementally refined and straightened their migration routes (Fig. 4). When comparing paired segments of the routes where individuals deviated from their past movements, these deviations were more direct ways to move between destinations during the spring migration (Fig. 4*B*). In resident animals, innovations of shortcuts between key destinations can be interpreted as evidence of a cognitive map used for navigation (29, 30), but only when alternative hypotheses like social facilitation or differences in environmental conditions are ruled out. Because we lacked data on group composition of our tagged storks, we are unable to rule out the possibility that social facilitation was responsible for innovating shortcuts during migration. In comparison to other tracked storks, individual routes became increasingly unique after age one and fidelity increased with age (Fig. 3), suggesting a switch from relying more on personal information instead of social information during later stages of life. Likewise, faster and earlier spring migrations of older birds (Fig. 1 and *SI Appendix*, Fig. S1) indicate that older individuals are likely leaders, not followers. A switch from reliance on social learning to experiential learning has been linked to the timing of migration in reintroduced whooping cranes [*Grus americana* (48)], while experienced homing pigeons (*Columba livia*) are more likely to lead during route finding (49). Further research is needed to determine whether the shortcutting we document is the result of social transmission of more direct movement paths or large-scale cognitive maps, similar to those discovered in bats. Regardless of the exact mechanism responsible for the innovation of spring migration shortcuts, the incremental straightening of the migration route over time is evidence of a switch from exploration to exploitation, which highlights the critical role of spatial memory in the ontogeny of migration (26, 27).

Learning is also critical in shaping the efficiency of migratory flight. Consistent with previous work, we found that compensation for wind drift was important in shaping migration routes (50). Young individuals were more likely to exhibit low route fidelity when exposed to strong crosswinds, but the influence of crosswinds diminished as storks aged and gained more experience (*SI Appendix*, Figs. S2 and S8). Furthermore, we found that the importance of environmental conditions extended to influence decisions about when to fly or remain stationary during spring migration. Specifically, choices about when to fly shifted toward increasing wind support and lowering exposure to crosswinds as birds aged, but these choices came at the expense of experiencing lower thermal uplift during flight (*SI Appendix*, Fig. S4). This suggests that older birds face a trade-off between favorable winds and favorable uplift during spring migration. Although we found some support for the notion that environmental conditions shaped the ontogeny of route fidelity, we were unable to detect an ontogenetic influence of environmental conditions on migration timing or flight energetics (*SI Appendix*, Fig. S2). We suspect this could be explained by two complementary processes. First, birds of all ages avoid flying when crosswinds where strong (*SI Appendix*, Figs. S3 and S4). Second, there is high selective pressure to learn to cope with adverse conditions early in life, potentially during the first fall migration (20). In fact, learning to cope with challenging flight conditions could be an early quality filter, where young storks (<2 y old) of poorer quality are more likely to die because of their inferior skills in coping with strong crosswinds or weak thermal uplift (8, 50). Since our analysis excluded individuals that died before completing two consecutive fall migrations, we were unable to investigate the existence of an early-life quality filter. Because few studies track the development of migration from early life onward, further research across migrants exhibiting different life histories and flight modalities is needed to investigate the generalizability of our findings to other species and systems.

Migration is often conceptualized as being underpinned by either genetic or culturally inherited information, yet emerging research is calling this simple dichotomization into question (10, 25). Whether the first migration is guided by genetics or results from following informed individuals, learning within a lifetime represents an additional and complementary mechanism shaping animal migration (10). There is evidence that even when the initial migration direction appears genetically encoded, increased experience and learning can improve migratory performance. For example, in several bird species, adults who had performed multiple previous migrations were better able to correct for large-scale displacements in comparison to juveniles during migration (51, 52), highlighting the importance of both genetics and individual learning. Furthermore, recent work has discovered the importance of genes related to the formation of long-term memory in migratory peregrine falcons [*Falco peregrinus* (53)], suggesting an important interplay between genetics, spatial memory, and learning for long-distance migration (54). Learning, by definition, involves the use of information to improve future performance on a task (26). Thus, the incremental refinements in migration timing and routes over a lifetime by white storks indicate that information is a key, but often overlooked and rarely quantified, currency shaping animal migration.

## Materials and Methods

### Early-Life Tracking Data.

We compiled tracking data from five study areas in Germany and Austria, where a total of 258 juvenile white storks were tagged and tracked from 2013 to 2021. Permits for tagging and tracking the white storks were issued by the authorities of the Federal States (G-13/28 and G-15/47 by Regierungspräsidium Freiburg, 54-2532.1-14/14 by Regierung von Mittelfranken, MPI-O-1/14 by Regierungspräsidium Tübingen, G15-20-032 by LUA Rheinland-Pfalz, and ROB-55.2Vet-2532.Vet_02-17-95 by Regierung von Oberbayern). Juvenile white storks were tagged on the nest with solar-powered GSM-GPS-ACC loggers that are programmed and designed to collect movement and activity data indefinitely (e-obs GmbH, for details, see ref. 55). All devices recorded GPS locations and three‐dimensional body acceleration for 18 h a day (between 2:00 and 20:00 UTC). GPS positions were recorded at intervals ranging from 1 s to 20 min; three‐axial body acceleration data were collected in short bursts (lengths ranging from 1.2 to 4.1 s) every 0.5 to 10 min at 10.54 to 33.33 Hz. Data were stored on the device until downloaded via an ultra‐high‐frequency radio link or sent via the mobile phone network.

We only included individuals that completed 2 or more spring and fall migrations along the Western European flyway, which resulted in 40 individuals, 190 animal-years, and 301 migration events (i.e., unique combinations of migration season, year, and individual) retained for further analysis (see *SI Appendix*, Table S2 for details). The GPS sampling schedules were slightly different across time and study areas, so we subsampled the GPS data to a consistent, 3-h sampling interval. These subsampled data were used for all subsequent analyses related to migration timing, route-level metrics, and extraction of environmental data on wind support and uplift strength.

### Quantifying Migration Timing.

We used Net Squared Displacement (NSD) to manually identify the start and end of each migration event (i.e., for each season and year that an individual was tracked), following the methods of ref. 56. NSD calculates the squared Euclidean distance between a reference location (in this case the initial nest site of each tagged juvenile) and subsequent relocations (54). Seasonal increases or decreases in NSD that occur rapidly and consecutively are indicative of highly directed, long-distance migration events (57). In addition to identifying migration start and end dates using NSD, we examined the spatial location of each identified start and end of migration to verify the selection (56). Migration duration was calculated as the difference, in days, between the end and start of each migration event.

### Flight Energetics.

Although white storks stop over and refuel during migration, which affects net energy balance, we focused our analysis solely on the energetics of flight because it can be reliably estimated using ACC data (45). We used three-axial ACC data collected from a subsample of tagged individuals (82.7% of animal-years) to estimate total energetic expenditure during migratory flight (43). We first identified periods of migratory flight as time windows when an individual moved greater than 2 m/s (20). For any raw ACC burst collected during periods of migratory flight, we converted raw ACC values in millivolts to units of gravitational acceleration (*g*) using tag-specific calibration values (55). We used established methods to calculate ODBA (45, 58), by first applying a running mean over each of the three ACC axes (x, y, and z) to generate smoothed time series. Then, the difference between smoothed values and unsmoothed values for each axis was calculated and summed to calculate ODBA (45, 58). The sampling duration and rate of ACC bursts varied slightly across study areas and time periods when tags were deployed. Thus, to yield comparable ODBA values, we first calculated the daily average ODBA values during migratory flight and then took the sum of those daily values during the migration period to calculate cumulative ODBA to estimate the total energetic expenditure during each migration event.

### Characterizing Migration Routes.

We used several route-level metrics to approximate exploration and exploitation during migration. We calculated migration distance as the great circle distance between the start and end points of each migration event. We calculated the straightness of a trajectory by dividing the Euclidean distance between the first and last point of the trajectory by the total length of the trajectory (59). Straightness was calculated over the entire migration route and for individual deviations from the previous year’s route (see below and *SI Appendix*, Fig. S9 for details). We used the trajectory similarity metric, DTW, to estimate route fidelity. DTW values range from 0 to infinity, with increasing values indicating lower fidelity (or increasing route dissimilarity). DTW is calculated by finding the minimum distance path (i.e., the warping path) between pairs of points in the trajectories being compared (47). We calculated DTW between all pairs of previous (t-1) and current (t) migrations for each individual and season.

To determine whether storks were incrementally refining their migration routes by flying more directly between destinations during migration, we identified paired segments of the migration route where movement paths deviated across years but had similar start and end locations (see *SI Appendix*, Fig. S9 for an example). To identify these paired deviations, we first calculated the movement corridor for each individual migration event as the 99% contour from the utilization distribution of a Brownian Bridge Movement Model (BBMM) (60). The BBMM utilization distribution was calculated on an identical raster grid (pixel resolution = 1 km^2^, spatial extent = the maximum spatial extent of all migration events, buffered by 10%). Route deviations for the current year were identified as sequences of at least four consecutive relocations that fell outside of the previous year’s corridor (which is equivalent to a minimum 12-h period spent outside of the previous year’s migration corridor; *SI Appendix*, Fig. S9). The corresponding previous year’s route deviation was identified as the series of points between locations from the previous year that were nearest (in distance) between the first and last point of the current year’s deviation (*SI Appendix*, Fig. S9).

### Statistical Analysis.

We were interested in understanding the importance of time, energy, and information as individuals aged. To estimate this, we built linear mixed-effects models to estimate the effect size and significance (i.e., if the estimated 95% CIs did not overlap zero) of age on migration duration, cumulative ODBA, route fidelity, and migration distance. Each model included random intercepts and slopes to account for repeated measures of the same individual over time. To control for potential confounders, we included variables known to impact migration, such as season (spring or fall migration), wind support, crosswinds, and uplift strength (9, 46, 55). Variables needed to calculate wind support, crosswinds, and uplift strength were extracted using the movebank.org Env-Data track annotation tool (61) from the European Centre for Medium-Range Weather Forecasts’ ERA-5 reanalysis model (spatial and temporal resolution of 30 km and 1 h, respectively). Specifically, wind support estimates the length of the wind vector (m/s) in the direction of the animal’s movement, while crosswinds estimate the length of the wind vector (m/s) in the direction perpendicular to the animal’s movement direction. Both wind support and crosswinds were calculated using established methods described in ref. 16. Since we were interested in the magnitude and not the direction of the crosswinds, we took the absolute value of crosswinds before calculating an average for each migration event. In our comparison of environmental conditions during flight and nonflight periods, we calculated wind support and crosswinds during stationary periods using the most recent movement heading at the animal’s current location. Uplift strength was estimated using vertical velocity of pressure (Pa s^−1^) following the approach described in ref. 31. Negative vertical velocity values represent upward air movement and positive values represent downward air movement. We transformed variables with skewed distributions before they were used in any models. The 95% CIs for all fixed effects included in the model were estimated using semiparametric bootstrapping (n = 1,000 simulations) using the bootMer function in the lme4 package (v 1.1-31) of R version 4.2.2 (62). The versions of all R packages used for analysis can be found in *SI Appendix*, Table S15.

To rule out the possible alternative explanation that the changes in migration characteristics occurred because of selective mortality, we reran all of the linear mixed-effects models excluding any animals that died during the study duration. We found no significant difference in coefficient estimates in any of the models (as indicated by overlap between the 95% CIs), which suggests that selective mortality had no obvious impact on our results (*SI Appendix*, Fig. S11). Our fidelity analysis required at least 2 y of data, thus initially including only animals that lived to be at least two years old likely minimized the potential impact of selective mortality.

We also examined whether there was any change in the directness of the entire migration route and route deviations between consecutive migration events. The overall difference in means in the straightness of previous and current routes or paired route deviations was estimated during fall and spring migration events using paired *t*-tests. A negative difference in means would indicate an overall straightening of movement trajectories across years.

## Supplementary Material

Appendix 01 (PDF)

## Data Availability

GPS tracking data and ACC data: Data from: Study “LifeTrack White Stork Oberschwaben” (2014–March 2023); Data from: Study “LifeTrack White Stork Vorarlberg” (2016–March 2023); Data from: Study “LifeTrack White Stork SW Germany” (2013–March 2023); Data from: Study “LifeTrack White Stork Rheinland-Pfalz” (2015–March 2023); Data from: Study “LifeTrack White Stork Bavaria” (2014–March 2023). Data have been deposited in Movebank (https://doi.org/10.5441/001/1.c42j3js7_2; https://doi.org/10.5441/001/1.71r7pp6q_2; https://doi.org/10.5441/001/1.ck04mn78_2; https://doi.org/10.5441/001/1.4192t2j4_2; https://doi.org/10.5441/001/1.v1cs4nn0_2) (34–38).

## References

[1] N. Tinbergen, On aims and methods of ethology. Zeitschrift Für Tierpsychologie **20**, 410–433 (1963).

[2] W. Xu , The plasticity of ungulate migration in a changing world. Ecology **102**, e03293 (2021). 33554353 10.1002/ecy.3293

[3] E. O. Aikens, I. D. Bontekoe, L. Blumenstiel, A. Schlicksupp, A. Flack, Viewing animal migration through a social lens. Trends Ecol. Evol. **37**, 985–996 (2022). 35931583 10.1016/j.tree.2022.06.008

[4] C. Buchan, J. J. Gilroy, I. Catry, A. M. A. Franco, Fitness consequences of different migratory strategies in partially migratory populations: A multi-taxa meta-analysis. J. Anim. Ecol. **89**, 678–690 (2020). 31777950 10.1111/1365-2656.13155 PMC7078763

[5] J. M. Fryxell, J. Greever, A. R. E. Sinclair, Why are migratory ungulates so abundant. Am. Nat. **131**, 781–798 (1988).

[6] W. W. Deacy , Phenological synchronization disrupts trophic interactions between Kodiak brown bears and salmon. Proc. Natl. Acad. Sci. U.S.A. **114**, 10432–10437 (2017). 28827339 10.1073/pnas.1705248114 PMC5625906

[7] S. Bauer, B. J. Hoye, Migratory animals couple biodiversity and ecosystem functioning worldwide. Science **344**, 1242552 (2014). 24700862 10.1126/science.1242552

[8] F. Sergio , Individual improvements and selective mortality shape lifelong migratory performance. Nature **515**, 410–413 (2014). 25252973 10.1038/nature13696

[9] A. Flack , New frontiers in bird migration research. Curr. Biol. **32**, R1187–R1199 (2022). 36283388 10.1016/j.cub.2022.08.028

[10] A. L. Fayet, Exploration and refinement of migratory routes in long-lived birds. J. Anim. Ecol. **89**, 16–19 (2020). 32091641 10.1111/1365-2656.13162

[11] T. Alerstam, Optimal bird migration revisited. J. Ornithol. **152**, 5–23 (2011).

[12] A. Hedenström, Migration by soaring or flapping flight in birds: The relative importance of energy cost and speed. Philos. Trans. R. Soc. B Biol. **342**, 353–361 (1993).

[13] T. Alerstam, Å. Lindström, “Optimal bird migration: The relative importance of time, energy, and safety” in Bird Migration, E. Gwinner, Ed. (Springer, Berlin, Heidelberg, 1990), pp. 331–351, 10.1007/978-3-642-74542-3_22.

[14] A. Hedenström, Adaptations to migration in birds: Behavioural strategies, morphology and scaling effects. Philos. Trans. R. Soc. B Biol. Sci. **363**, 287–299 (2008). 10.1098/rstb.2007.2140 PMC2606751 17638691

[15] A. M. Hein, C. Hou, J. F. Gillooly, Energetic and biomechanical constraints on animal migration distance. Ecol. Lett. **15**, 104–110 (2012). 22093885 10.1111/j.1461-0248.2011.01714.x

[16] K. Safi , Flying with the wind: Scale dependency of speed and direction measurements in modelling wind support in avian flight. Movement Ecol. **1**, 4 (2013). 10.1186/2051-3933-1-4 PMC4337751 25709818

[17] H. Kokko, Competition for early arrival in migratory birds. J. Anim. Ecol. **68**, 940–950 (1999).

[18] E. L. C. Shepard , Energy landscapes shape animal movement ecology. Am. Nat. **182**, 298–312 (2013). 23933722 10.1086/671257

[19] H. J. Williams , Physical limits of flight performance in the heaviest soaring bird. Proc. Natl. Acad. Sci. U.S.A. **117**, 17884–17890 (2020). 32661147 10.1073/pnas.1907360117 PMC7395523

[20] S. Rotics , The challenges of the first migration: Movement and behaviour of juvenile vs. adult white storks with insights regarding juvenile mortality. J. Anim. Ecol. **85**, 938–947 (2016). 27046512 10.1111/1365-2656.12525

[21] K. Yoda , Compass orientation drives naïve pelagic seabirds to cross mountain ranges. Curr. Biol. **27**, R1152–R1153 (2017). 29112864 10.1016/j.cub.2017.09.009

[22] M. Hake, N. Kjellén, T. Alerstam, Age-dependent migration strategy in honey buzzards Pernis apivorus tracked by satellite. Oikos **103**, 385–396 (2003).

[23] C. S. Teitelbaum , Experience drives innovation of new migration patterns of whooping cranes in response to global change. Nat. Commun. **7**, 12793 (2016). 27597446 10.1038/ncomms12793 PMC5025849

[24] Y. Cheng, W. Fiedler, M. Wikelski, A. Flack, “Closer-to-home” strategy benefits juvenile survival in a long-distance migratory bird. Ecol. Evol. **9**, 8945–8952 (2019). 31462993 10.1002/ece3.5395 PMC6706183

[25] L. Campioni, M. P. Dias, J. P. Granadeiro, P. Catry, An ontogenetic perspective on migratory strategy of a long-lived pelagic seabird: Timings and destinations change progressively during maturation. J. Anim. Ecol. **89**, 29–43 (2020). 31206644 10.1111/1365-2656.13044

[26] M. A. Lewis , Learning and animal movement. Front. Ecol. Evol. **9**, 681704 (2021).

[27] W. F. Fagan , Spatial memory and animal movement. Ecol. Lett. **16**, 1316–1329 (2013). 23953128 10.1111/ele.12165

[28] T. Mueller, R. B. O’Hara, S. J. Converse, R. P. Urbanek, W. F. Fagan, Social learning of migratory performance. Science **341**, 999–1002 (2013). 23990559 10.1126/science.1237139

[29] L. Harten, A. Katz, A. Goldshtein, M. Handel, Y. Yovel, The ontogeny of a mammalian cognitive map in the real world. Science **369**, 194–197 (2020). 32647001 10.1126/science.aay3354

[30] S. Toledo , Cognitive map-based navigation in wild bats revealed by a new high-throughput tracking system. Science **369**, 188–193 (2020). 32647000 10.1126/science.aax6904

[31] H. Brønnvik, K. Safi, W. M. G. Vansteelant, P. Byholm, E. Nourani, Experience does not change the importance of wind support for migratory route selection by a soaring bird. R. Soc. Open Sci. **9**, 220746 (2022). 36569232 10.1098/rsos.220746 PMC9768468

[32] C. K. Frankish, A. Manica, T. A. Clay, A. G. Wood, R. A. Phillips, Ontogeny of movement patterns and habitat selection in juvenile albatrosses. Oikos **2022**, e09057 (2022).

[33] T. Guilford , A dispersive migration in the Atlantic Puffin and its implications for migratory navigation. PLoS One **6**, e21336 (2011). 21799734 10.1371/journal.pone.0021336 PMC3140476

[34] W. Fiedler, A. Flack, A. Schmid, U. Reinhard, M. Wikelski, Data from: Study “LifeTrack White Stork Oberschwaben” (2014-2023). Movebank Data Repository. 10.5441/001/1.c42j3js7_2. Deposited 17 December 2023.

[35] W. Fiedler, W. Niederer, A. Schönenberger, A. Flack, M. Wikelski, Data from: Study “LifeTrack White Stork Vorarlberg” (2016-2023). Movebank Data Repository. 10.5441/001/1.71r7pp6q_2. Deposited 17 December 2023.

[36] W. Fiedler , Data from: Study “LifeTrack White Stork SW Germany” (2013-2023). Movebank Data Repository. 10.5441/001/1.ck04mn78_2. Deposited 17 December 2023.

[37] W. Fiedler , Data from: Study “LifeTrack White Stork Rheinland-Pfalz” (2015-2023). Movebank Data Repository. 10.5441/001/1.4192t2j4_2. Deposited 17 December 2023.

[38] W. Fiedler , Data from: Study “LifeTrack White Stork Bavaria” (2014-2023). Movebank Data Repository. 10.5441/001/1.v1cs4nn0_2. Deposited 17 December 2023.

[39] I. D. Bontekoe, R. Hilgartner, W. Fiedler, A. Flack, The price of being late: Short- and long-term consequences of a delayed migration timing. Proc. R. Soc. B Biol. Sci. **290**, 20231268 (2023). 10.1098/rspb.2023.1268 PMC10369029 37491964

[40] G. Creutz, Der Weissstorch: Ciconia ciconia (A. Ziemsen, 1988), vol. 375.

[41] C. J. Pennycuick, Modelling the Flying Bird (Elsevier, 2008).

[42] O. Duriez , How cheap is soaring flight in raptors? A preliminary investigation in freely-flying vultures. PLoS One **9**, e84887 (2014). 24454760 10.1371/journal.pone.0084887 PMC3893159

[43] P. Vergara, O. Gordo, J. I. Aguirre, Nest size, nest building behaviour and breeding success in a species with nest reuse: The white stork *Ciconia ciconia*. Ann. Zool. Fenn. **47**, 184–194, 111 (2010).

[44] M. Tobolka, S. Kuźniak, K. M. Zolnierowicz, T. H. Sparks, P. Tryjanowski, New is not always better: Low breeding success and different occupancy patterns in newly built nests of a long-lived species, the white stork Ciconia ciconia. Bird Study **60**, 399–403 (2013).

[45] R. P. Wilson , Moving towards acceleration for estimates of activity-specific metabolic rate in free-living animals: The case of the cormorant. J. Anim. Ecol. **75**, 1081–1090 (2006). 16922843 10.1111/j.1365-2656.2006.01127.x

[46] A. Flack, M. Nagy, W. Fiedler, I. D. Couzin, M. Wikelski, From local collective behavior to global migratory patterns in white storks. Science **360**, 911–914 (2018). 29798883 10.1126/science.aap7781

[47] I. R. Cleasby , Using time-series similarity measures to compare animal movement trajectories in ecology. Behav. Ecol. Sociobiol. **73**, 151 (2019).

[48] B. Abrahms, C. S. Teitelbaum, T. Mueller, S. J. Converse, Ontogenetic shifts from social to experiential learning drive avian migration timing. Nat. Commun. **12**, 7326 (2021). 34916500 10.1038/s41467-021-27626-5 PMC8677782

[49] A. Flack, B. Pettit, R. Freeman, T. Guilford, D. Biro, What are leaders made of? The role of individual experience in determining leader–follower relations in homing pigeons. Anim. Behav. **83**, 703–709 (2012).

[50] F. Sergio , Compensation for wind drift during raptor migration improves with age through mortality selection. Nat. Ecol. Evol. **6**, 989–997 (2022). 35680999 10.1038/s41559-022-01776-1

[51] S. Åkesson, H. Bakam, E. Martinez Hernandez, M. Ilieva, G. Bianco, Migratory orientation in inexperienced and experienced avian migrants. Ethol. Ecol. Evol. **33**, 206–229 (2021).

[52] K. Thorup , Evidence for a navigational map stretching across the continental US in a migratory songbird. Proc. Natl. Acad. Sci. U.S.A. **104**, 18115–18119 (2007). 17986618 10.1073/pnas.0704734104 PMC2084305

[53] Z. Gu , Climate-driven flyway changes and memory-based long-distance migration. Nature **591**, 259–264 (2021). 33658718 10.1038/s41586-021-03265-0

[54] S. Lisovski, M. Liedvogel, A bird’s migration decoded. Nature **591**, 203–204 (2021). 33658671 10.1038/d41586-021-00510-4

[55] A. Flack , Costs of migratory decisions: A comparison across eight white stork populations. Sci. Adv. **2**, e1500931 (2016). 26844294 10.1126/sciadv.1500931 PMC4737271

[56] E. O. Aikens , The greenscape shapes surfing of resource waves in a large migratory herbivore. Ecol. Lett. **20**, 741–750 (2017). 28444870 10.1111/ele.12772

[57] N. Bunnefeld , A model-driven approach to quantify migration patterns: Individual, regional and yearly differences. J. Anim. Ecol. **80**, 466–476 (2011). 21105872 10.1111/j.1365-2656.2010.01776.x

[58] A. C. Gleiss, R. P. Wilson, E. L. C. Shepard, Making overall dynamic body acceleration work: On the theory of acceleration as a proxy for energy expenditure. Methods Ecol. Evol. **2**, 23–33 (2011).

[59] S. Benhamou, How to reliably estimate the tortuosity of an animal’s path: Straightness, sinuosity, or fractal dimension? J. Theor. Biol. **229**, 209–220 (2004). 15207476 10.1016/j.jtbi.2004.03.016

[60] H. Sawyer, M. J. Kauffman, R. M. Nielson, J. S. Horne, Identifying and prioritizing ungulate migration routes for landscape-level conservation. Ecol. Appl. **19**, 2016–2025 (2009). 20014575 10.1890/08-2034.1

[61] S. Dodge , The environmental-data automated track annotation (Env-DATA) system: Linking animal tracks with environmental data. Movement Ecol. **1**, 1–14 (2013). 10.1186/2051-3933-1-3 PMC4337772 25709817

[62] D. Bates , Package ‘lme4’. (2009), http://lme4.r-forge.r-project.org.

