# Supplementary material for: Learning shapes the development of migratory behavior
Source: Proc Natl Acad Sci U S A. 2024 Mar 4;121(12):e2306389121. doi: 10.1073/pnas.2306389121 (PMC10962998; doi:10.1073/pnas.2306389121)
Supplement: Supplementary file 1 — Appendix 01 (PDF) [file pnas.2306389121.sapp.pdf]

Supporting Information for

Learning shapes the development of migratory behavior

Ellen O. Aikens, Elham Nourani, Wolfgang Fiedler, Martin Wikelski, Andrea Flack

Correspondence to: Ellen O. Aikens or Andrea Flack
Email: eaikens@uwyo.edu or aflack@ab.mpg.de

This PDF file includes:

Figures S1 to S11
Tables S1 to S15

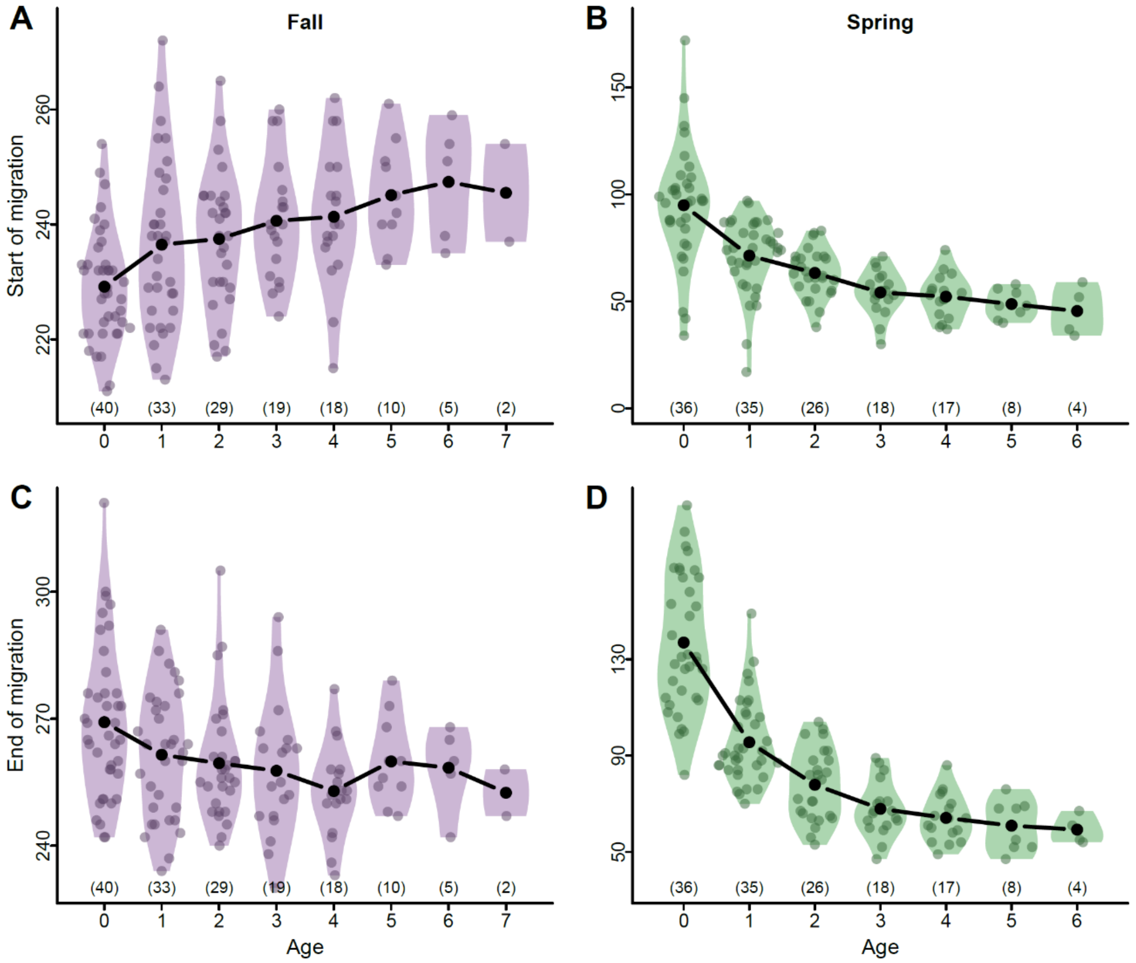

Fig S1. Changes in migration timing of white storks as they aged. Violin plots with an overlay of empirical points show the start of migration in Fall (A) and Spring (B), and the end of migration in Fall (C) and Spring (D). All start and end migration dates are displayed as the day of the year. Black dots show that mean value for each age class in A-D. Numbers in parentheses above the x-axis show the sample size for each age class.

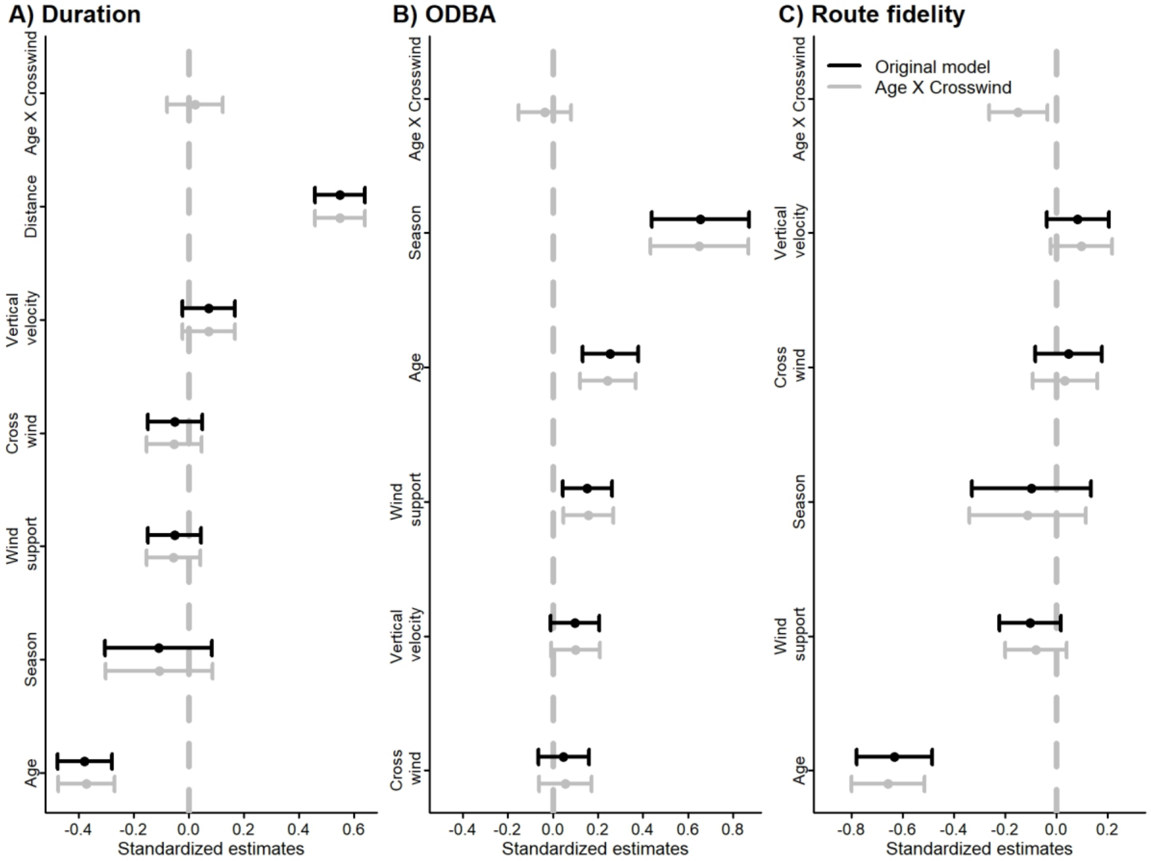

Fig S2. To examine the possibility that age-related differences in migration timing, flight energetics and route fidelity could be due to an increased ability of older and more experienced birds to compensate for adverse environmental conditions (i.e., strong crosswinds), we investigated how the inclusion of an interaction between crosswinds and age effected parameter estimates of models predicting migration duration (A), cumulative ODBA during migration (B) and migration route fidelity (as measured by Dynamic Time Warping; C). In all cases, except for route fidelity, including an interaction between age and crosswinds resulted in a large amount of overlap between the 95% CIs from the original models (black) and the models including the interaction (grey), suggesting that an increased ability of older birds to compensate for adverse conditions was not impacting age-related changes in migration duration or cumulative ODBA.

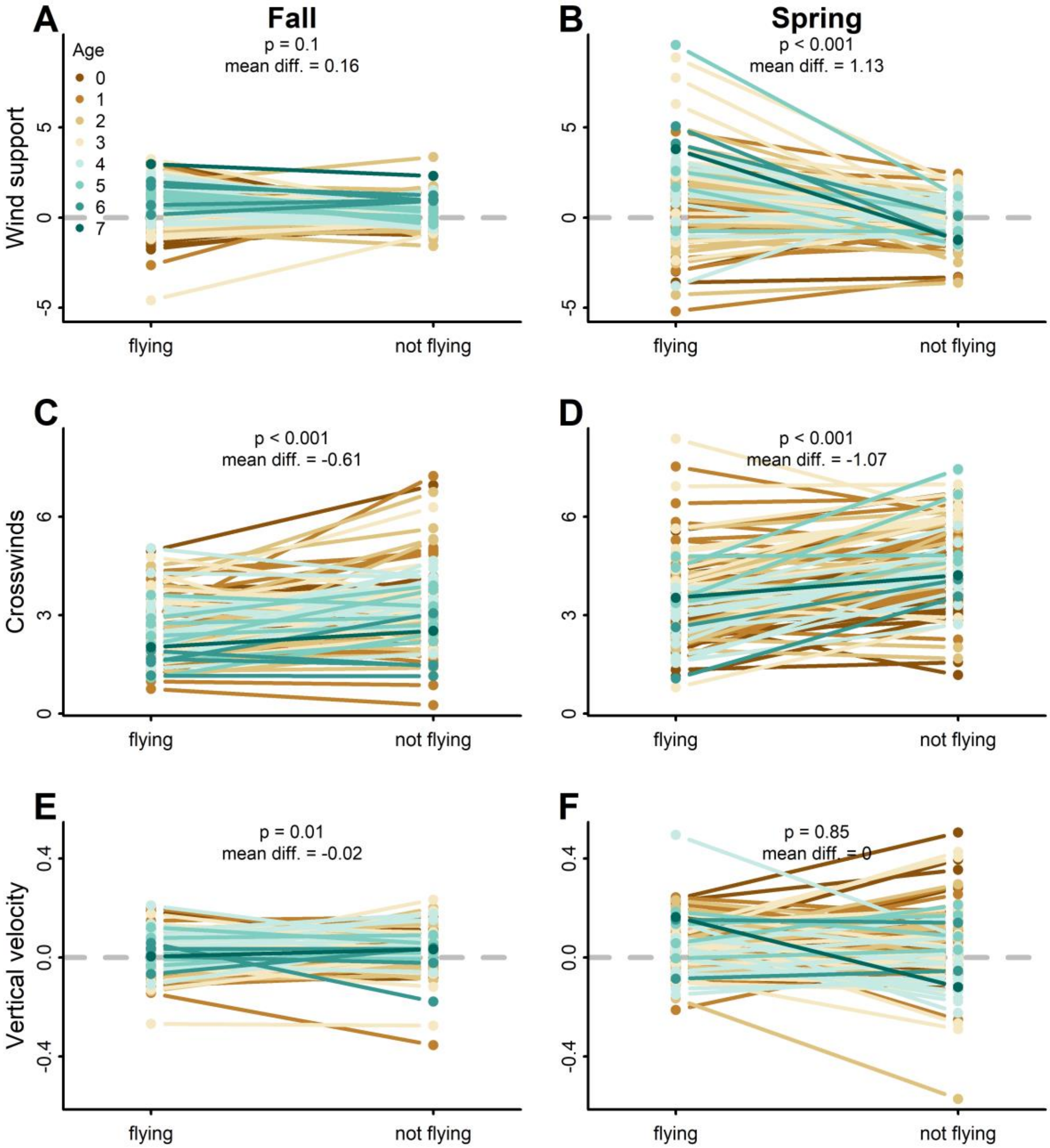

Fig S3. A comparison of environmental conditions experienced by white storks during flight and non-flight periods of Fall (A, C, E) and Spring (B, D, F) migration. We compared wind support (large values represent favorable flight conditions; A-B), crosswinds (small values represent favorable flight conditions; C-D) and vertical velocity (negative values represent upwards air movement and favorable flight conditions; E-F). We used two-sided paired t-tests to examine differences across flight and non-flight periods, where a positive difference in means represents larger values experienced during the flight period in comparison to the non-flight period for the environmental variable being considered (e.g., wind support, crosswinds, or vertical velocity).

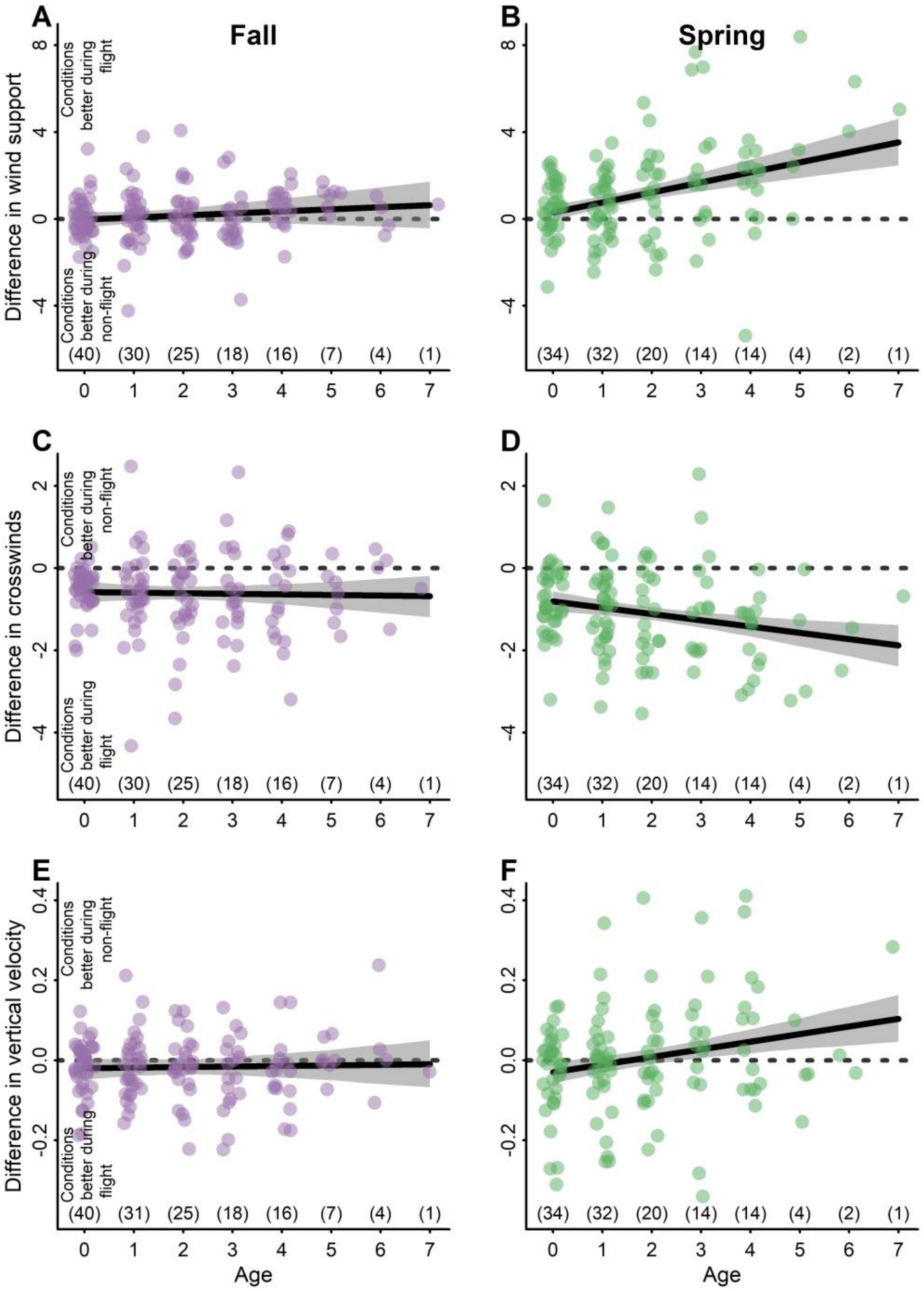

Fig S4. An examination of ontogenetic changes in decisions about when to fly or remain stationary during migration. We examined how differences in wind support (A-B), crosswinds (C-D) and vertical velocity (a proxy for uplift strength; E-F) experienced during flight and non-flight periods changed across seasons as animals aged. Larger, positive differences in wind support represent more favorable conditions during flight in comparison to non-flight periods, whereas smaller, negative differences in crosswinds and vertical velocity represent more favorable conditions during flight in comparison to non-flight periods. The numbers in parentheses above the x-axes indicate the sample size for each age class. The solid black line shows the fitted relationship and the grey polygon indicates the 95% CI estimated by semiparametric bootstrapping (n=1,000 simulations). n= 262 migration events (spring and fall migrations) from 40 individuals.

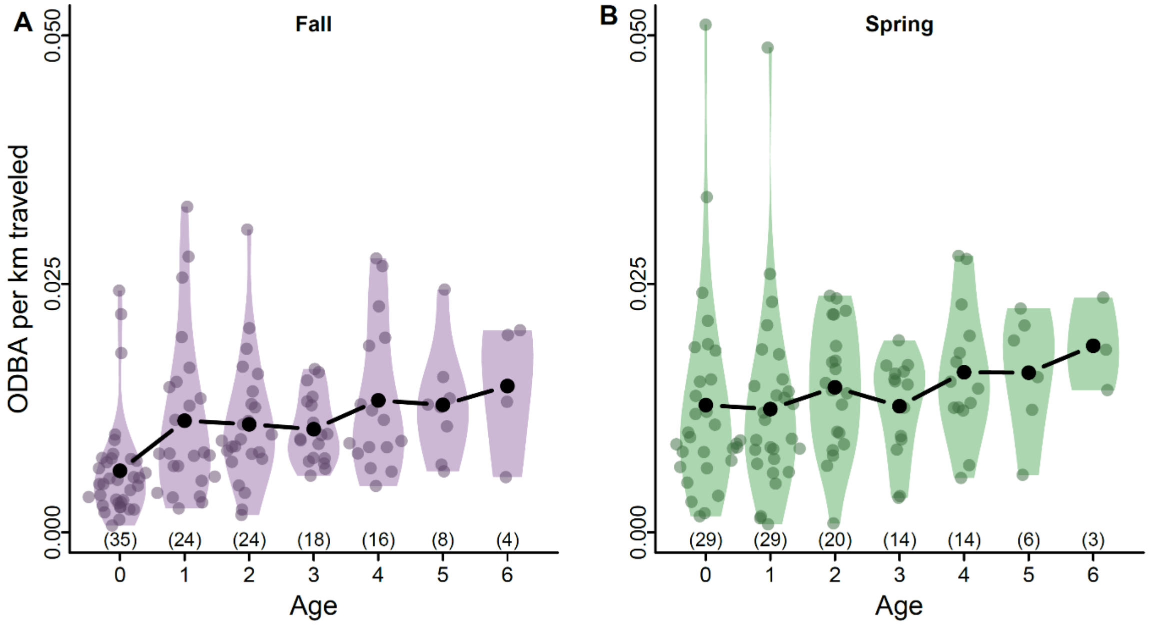

Fig S5. Changes in the Overall Dynamic Body Acceleration (ODBA) per distance traveled as individuals aged. ODBA was calculated only during migratory flight and increased with age during both Fall (A) and Spring (B) migration. Violin plots display the distribution for each age class and season with empirical data overlayed in each season-specific color (fall = purple, green = spring). Black dots show the mean ODBA per distance traveled for each age class. Numbers in parentheses show the sample size for each age class.

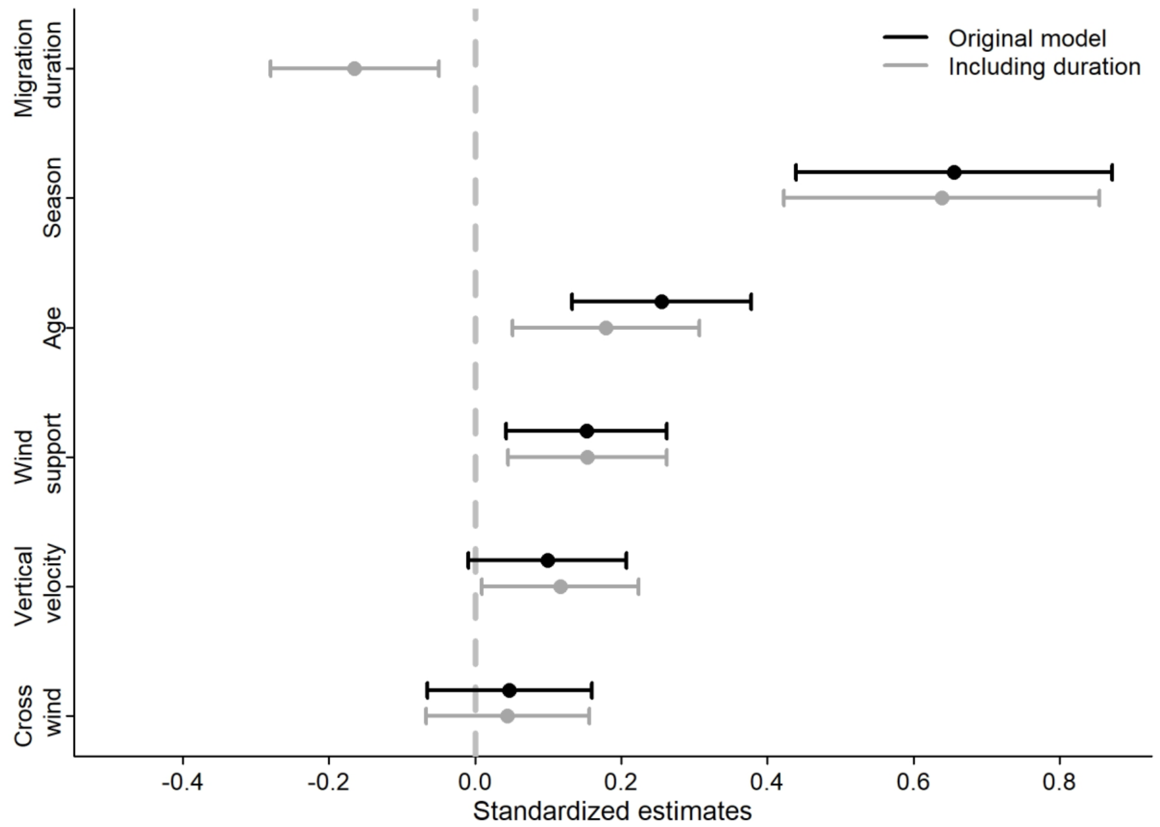

Fig S6. A comparison of coefficient estimates with 95% CIs derived for a model of cumulative ODBA during migratory flight that included age, environmental characteristics and season (black lines; hereafter the original model), and a model of cumulative ODBA including all variables in the original model with the addition of migration duration (grey lines). The addition of migration duration did not change the significance or effect size of any of the estimated coefficients (as indicated by overlap between the 95% CIs).

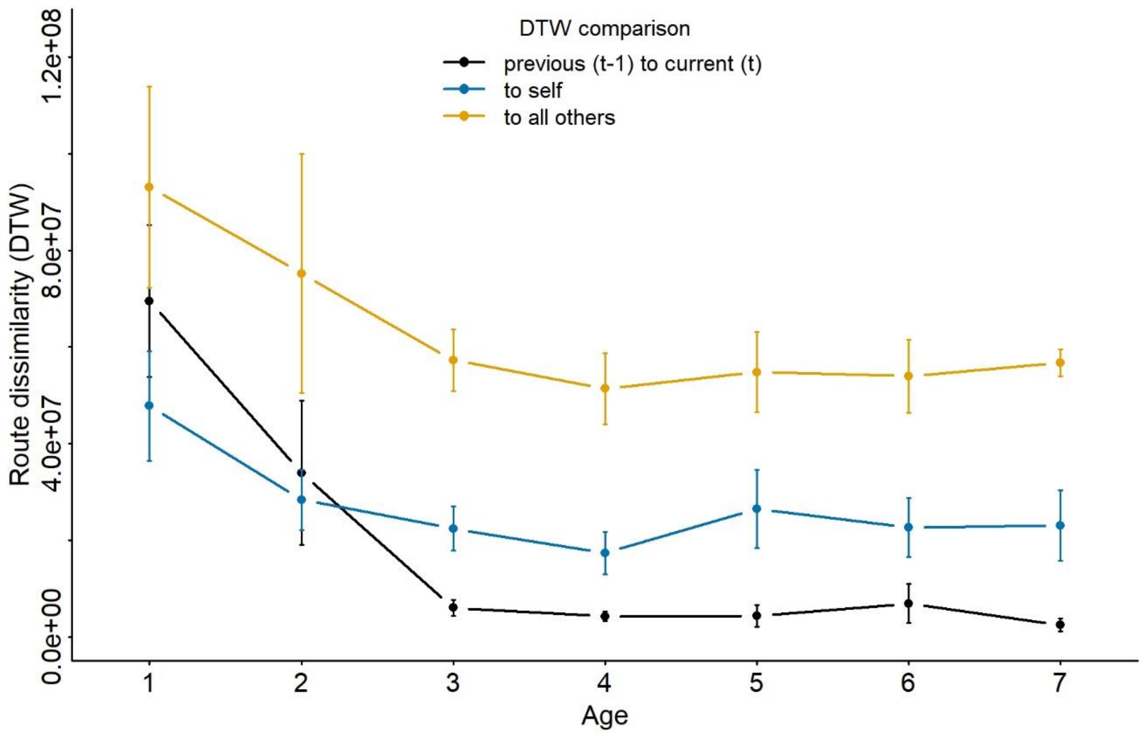

Fig S7. A comparison of Dynamic Time Warping (DTW) values between current migration events and events from the previous year (i.e., sequential comparisons; black), between current migration events and all other migration events from the same individual (i.e., “to self”; blue), and between current migration events and all migration events from all other individuals (i.e., “to all others”; orange). The average values with 95% CI for each age class and DTW comparison are shown. DTW is a trajectory similarity metric, with smaller values representing more similar trajectories (i.e., higher fidelity) and larger values representing more dissimilar trajectories (i.e., lower fidelity). We interpret the lack of overlap between the sequential comparisons and the “all others” comparison as evidence that animals are unlikely to be following others or converging on an optimal route used by the majority. Because there was overlap between sequential comparisons and all others comparisons at age one, this indicates that some social learning may occur in early life. However, the decline in DTW values for sequential comparisons suggests that routes become more individualized as the animal ages. Sequential comparisons and comparison to self overlap during ages 1-2, but diverge after age 2. This pattern indicates that exploration likely occurs before age 3, but after age 3 individuals tend to adhere to the migration route used in the previous year.

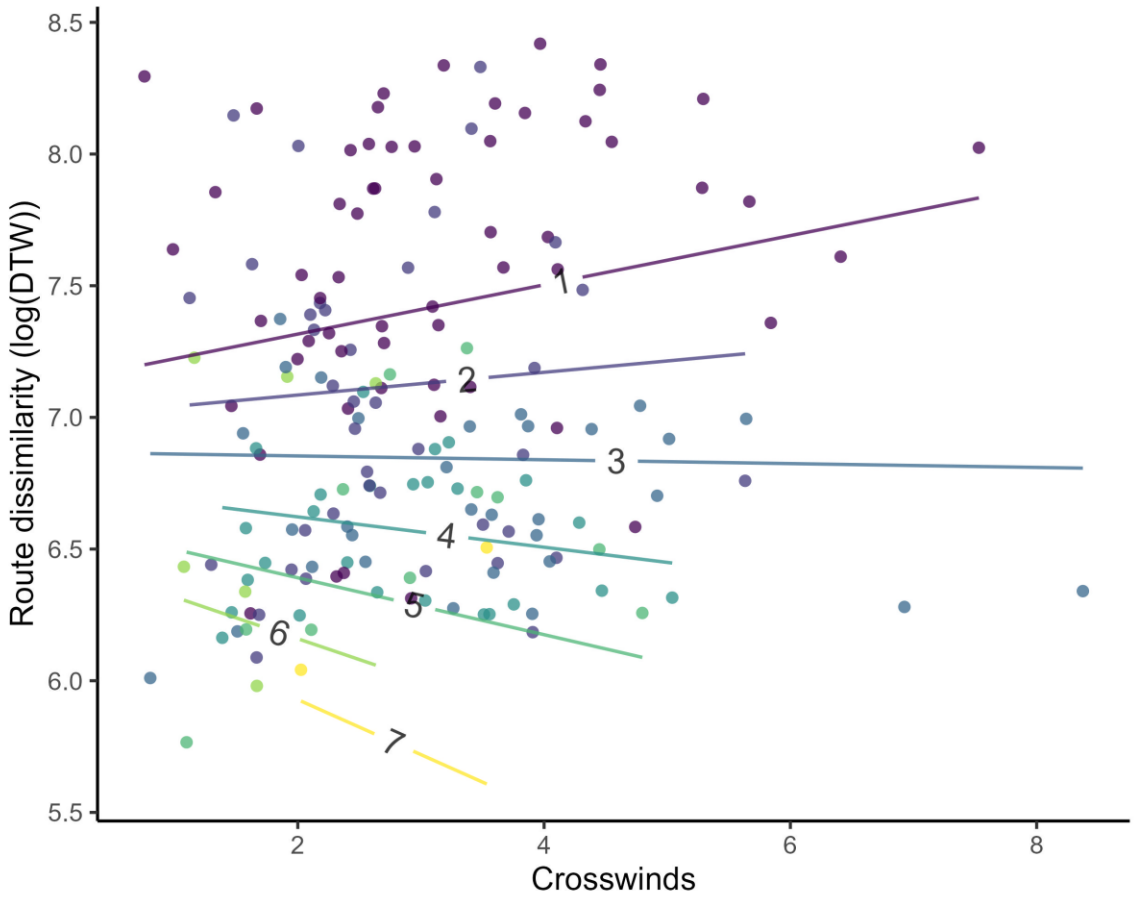

Fig S8. The interactive effect of age and crosswinds on route fidelity (see Table S12). Route fidelity was measured as the log of Dynamic Time Warping (DTW), where smaller values represent greater route fidelity. Crosswinds represent the absolute value of the wind vector perpendicular to the movement direction of storks during periods of migratory flight (where larger crosswinds represent more challenging conditions for flight). The color of each point represents the age of the individual (with the purple to yellow color gradient corresponding to ages 1 – 7).

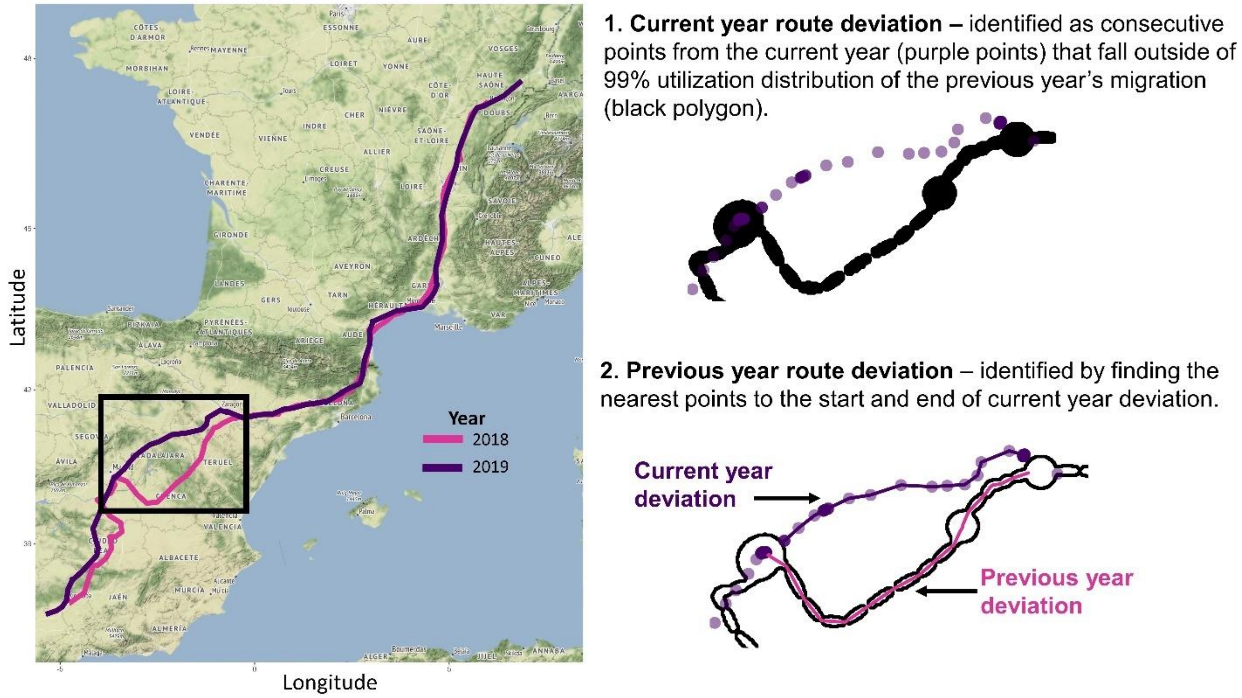

Fig S9. A visualization of the steps involved in identifying route deviations, using an example from the fall migrations of a white stork tracked in 2018 (pink) and 2019 (purple).

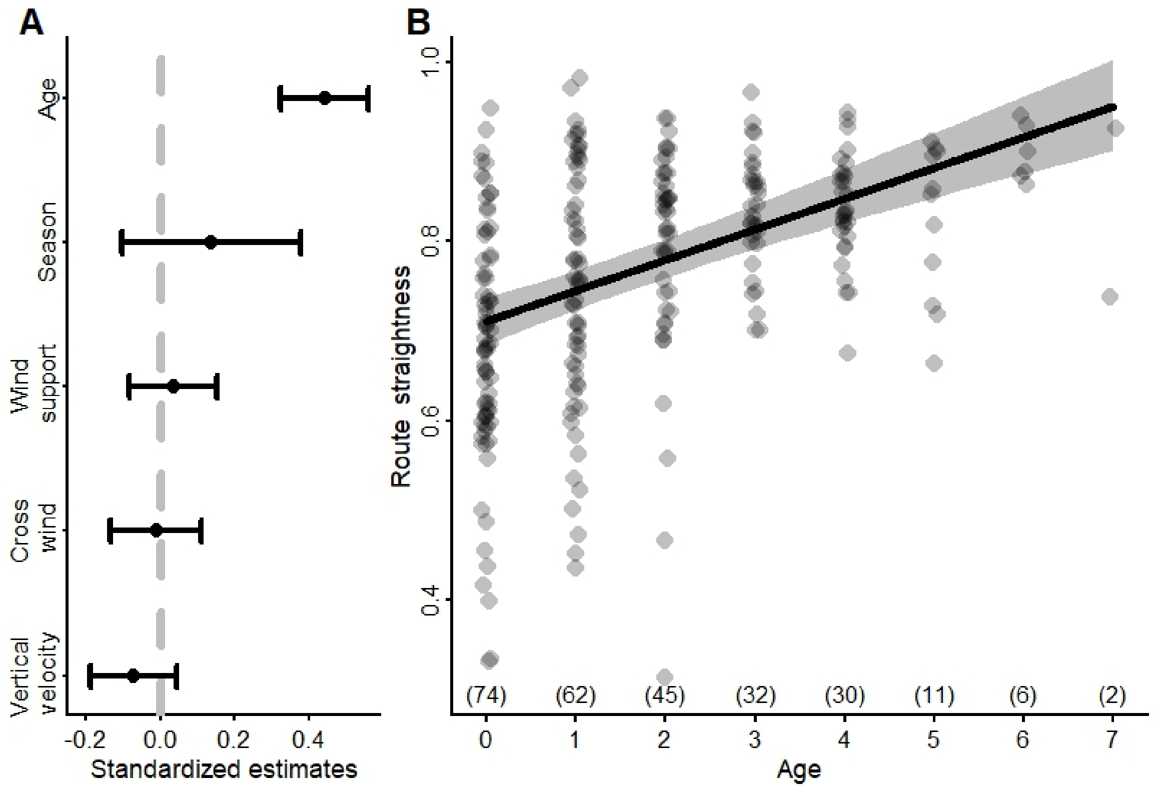

Fig S10. The influence of age on route straightness. (A) Standardized coefficient estimates of the fixed effects of a model examining the impact of age on straightness of the entire route, while controlling for potentially confounding effects of season and environmental conditions (i.e., wind support, crosswinds, and uplift strength estimated as vertical velocity). (B) As individuals aged, route straightness increased, suggesting that birds move more directly between migratory destinations as they age. A straightness value of one represents the most direct movement path between start and end points (i.e., the beeline). The numbers in parentheses above the x-axis in B indicate the sample size for each age class. In B, the solid black line shows the fitted relationship and the grey polygon indicates the 95% CI estimated by semiparametric bootstrapping (n=1,000 simulations). n= 262 migration events (spring and fall migrations) from 40 individuals.

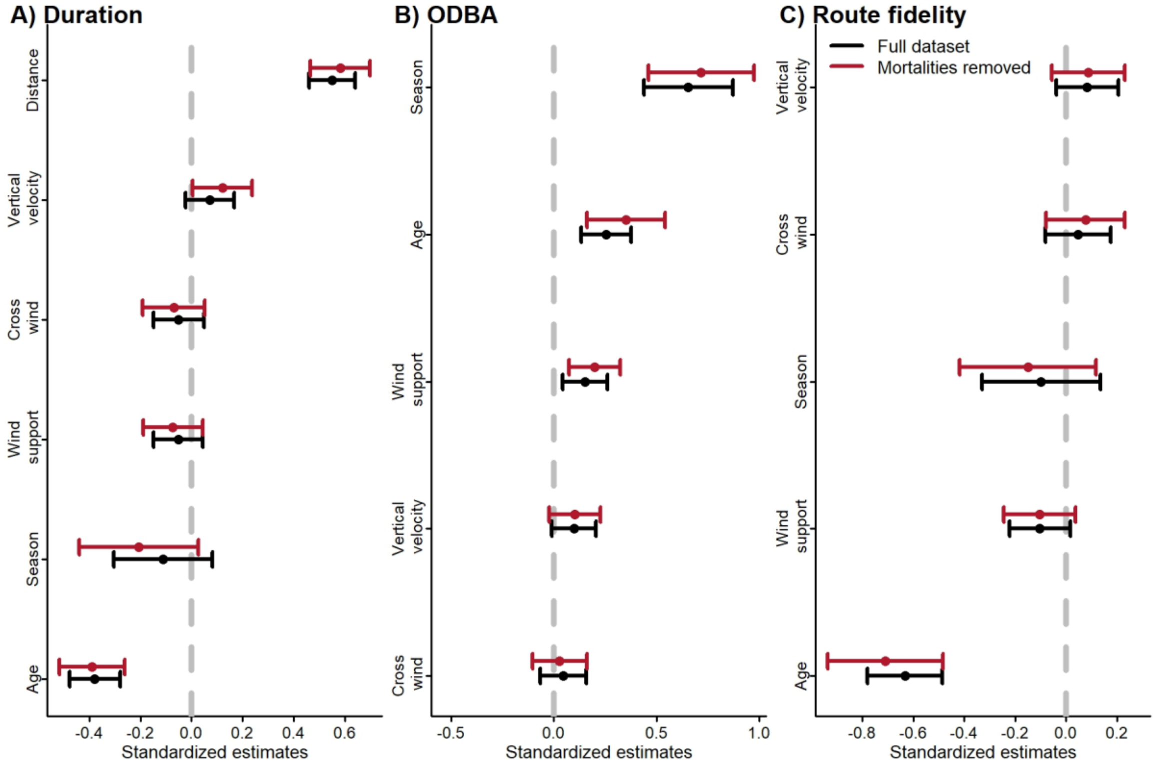

Fig S11. A comparison of coefficient estimates with 95% CIs derived from the full, original dataset (black) and the dataset with any mortalities that occurred after age two removed (dark red), to examine the potential impact of selective mortality on the estimated effect size. Because our fidelity analysis required at least two consecutive fall migrations, the original dataset already excluded any mortalities that occurred before that period. This comparison was made for A) the model estimating the impact of age on migration duration, B) the model estimating the effect of age on cumulative Overall Dynamic Body Acceleration (ODBA) and C) the model estimating the effect of age on route fidelity. In all cases, including or excluding individuals that died after age two had no impact on the estimated coefficients in any of the models (as indicated by the overlap between the 95% CIs). This indicates that selective mortality had no measurable effect on the patterns we observed and that learning is the most likely driver of changes in migration timing, energetics and route characteristics.

Table S1. The locations and sample sizes of the stork tagging areas.

**Region**	**Location**	**Number of individuals tracked**	**Number of migration events**
Bavaria, Germany	49.84° N, 10.82° E	7	45
Oberschwaben, Germany	48.01° N, 9.02° E	5	47
Rheinland-Pfalz, Germany	49.22° N, 8.17° E	14	83
Southwest Germany	47.75° N, 8.93° E	13	116
Vorarlberg, Austria	47.39° N, 9.70° E	1	10

Table S2. Details on the 40 individuals used in analysis, including the month and year they were tagged as fledglings, the date of death (if applicable), the status of the tag, the breeding population where they were tagged and the number of migration events (including spring and fall migrations) extracted from their tracking data.

Band ID	Date tagged (year-month)	Date of death (year-month-day)	Tag status	Population	N migration events
DER AU893	2016-06	2019-09-21		Bavaria	6
DER AU891	2016-06	NA	Transmitting in 2023	Bavaria	9
DER AU860	2015-06	NA	Transmitting in 2023	Bavaria	12
DER AT899	2015-06	2021-04-21		Bavaria	8
DER AT885	2014-06	2016-06-23		Bavaria	3
DER AT900	2015-06	2021-01-21		Bavaria	3
DER A2M38	2019-06	NA	Transmitting in 2023	Bavaria	4
DER AW838	2015-06	2017-09-29		Oberschwaben	4
DER AU640	2014-06	2019-10-08		Oberschwaben	11
DER AW844	2015-06	NA	Transmitting in 2023	Oberschwaben	12
DER AU650	2014-06	2022-02-06		Oberschwaben	14
DER AU599	2014-06	NA	Transmitting in 2023	Oberschwaben	6
DER AU829	2016-06	NA	Transmitting in 2023	Vorarlberg	10
DER AX142	2011-06	NA	Transmitting in 2023	Rheinland-Pfalz	10
DER AN444	2016-06	NA	Transmitting in 2023	Rheinland-Pfalz	10
DER A1R26	2018-06	NA (alive in 2023)	Malfunction Oct 2020	Rheinland-Pfalz	5
DER AW377	2018-06	2019-09-06		Rheinland-Pfalz	3
DER AX274	2017-06	2019-12-01		Rheinland-Pfalz	4
DER AX383	2016-06	2018-10-24		Rheinland-Pfalz	5

DER AW534	2018-06	NA	Transmitting in 2023	Rheinland-Pfalz	5
DER AN443	2016-06	NA	Transmitting in 2023	Rheinland-Pfalz	9
DER AT248	2015-06	NA (Seen alive in 2023)	Tag malfunction June 2019.	Rheinland-Pfalz	7
DER A1P74	2018-06	NA	Transmitting in 2023	Rheinland-Pfalz	6
DER A1P75	2018-06	NA	Transmitting in 2023	Rheinland-Pfalz	6
DER AX145	2016-06	2018-11-20		Rheinland-Pfalz	5
DER AW244	2016-06	2021-08-21		Rheinland-Pfalz	4
DER A3L55	2019-06	NA (seen alive in 2023)	Tag malfunction April 2021.	Rheinland-Pfalz	4
DER AY470	2016-06	NA	Transmitting in 2023	SW Germany	8
DER A1A26	2017-06	NA (seen alive in 2023)	Tag malfunction Mar 2023	SW Germany	8
DER AN909	2014-06	2016-04-01		SW Germany	3
DER AU050	2014-06	NA	Transmitting in 2023	SW Germany	14
DER AN910	2014-06	2020-09-22		SW Germany	12
DER AN922	2014-06	NA	Transmitting in 2023	SW Germany	7
DER AU053	2014-06	2017-06-01		SW Germany	6
DER AU057	2014-06	2019-03-27		SW Germany	10
DER AU017	2014-06	2020-04-02		SW Germany	10
DER AN858	2013-06	NA	Tag malfunction Dec 2021	SW Germany	16
DER AN918	2014-06	2016-05-01		SW Germany	4
DER AN861	2013-06	2014-12-14		SW Germany	3
DER AL581	2013-06	2021-02-24		SW Germany	15

Table S3. Statistical summary of the fixed effects of a linear mixed effects model examining the effect of age on migration duration while accounting for potentially confounding effects of migration distance (km, natural log transformed), season (Fall is the reference category), and environmental conditions (i.e., wind support, crosswinds, uplift). Random effects include a random intercept for individual ID and a random slope (Age|ID) to account for repeated measures on the same individual over time. In contrast to the standardized estimates shown in Figure 1, all units here are unstandardized. Model estimates are based on 262 migration events (spring and fall migrations) from 40 unique individuals.

**Coefficient**	**Estimate**	**95% CI**	**Standard Error**	**t value**
Intercept	-3.61	-4.77 – -2.46	0.59	-6.06
Age	-0.20	-0.26 – -0.15	0.027	-7.51
ln(Distance[km])	2.28	1.91 – 2.65	0.19	11.93
Wind support	-0.026	-0.073 – 0.021	0.024	-1.07
Crosswind	-0.040	-0.12 – 0.037	0.040	-1.01
Vertical velocity	0.72	-0.22 – 1.66	0.48	1.50
Season (Spring)	-0.10	-0.27 – 0.074	0.089	-1.13

Table S4. Statistical summary of the fixed effects of a linear mixed effects model examining the effect of age on the start of fall migration while accounting for potentially confounding effects of migration distance (calculated as the great circle distance between the start and end of migration), wind support, crosswinds, and vertical velocity (a proxy for uplift strength). Random effects include a random intercept for individual ID and a random slope (Age|ID) to account for repeated measures on the same individual over time. Model estimates are based on 141 fall migration events from 40 unique individuals.

**Coefficient**	**Estimate**	**95% CI**	**Standard Error**	**t value**
Intercept	288.45	260.71 – 316.94	12.80	22.54
Age	2.67	1.41 – 3.92	0.65	4.10
Migration distance	-19.34	-27.93 – -11.06	4.09	-4.72
Wind support	-0.53	-2.02 – 1.24	0.85	-0.63
Vertical velocity	-4.60	-24.86 – 34.84	13.60	-0.34
Crosswinds	0.76	-1.60 – 3.00	1.06	-0.71

Table S5. Statistical summary of the fixed effects of a linear mixed effects model examining the effect of age on the end of fall migration while accounting for potentially confounding effects of migration distance (calculated as the great circle distance between the start and end of migration), wind support, crosswinds, and vertical velocity (a proxy for uplift strength). Random effects include a random intercept for individual ID and a random slope (Age|ID) to account for repeated measures on the same individual over time. Model estimates are based on 141 fall migration events from 40 unique individuals.

**Coefficient**	**Estimate**	**95% CI**	**Standard Error**	**t value**
Intercept	194.95	160.87 – 231.31	18.13	10.75
Age	-1.68	-3.22 – -0.048	0.77	-2.16
Migration distance	23.10	12.47 – 33.90	5.82	3.97
Wind support	-2.44	-5.05 – -0.09	1.21	-2.01
Vertical velocity	22.39	-14.74 – 63.03	19.08	1.17
Crosswinds	-0.13	-3.67 – 3.06	1.56	-0.09

Table S6. Statistical summary of the fixed effects of a linear mixed effects model examining the effect of age on the start of spring migration while accounting for potentially confounding effects of migration distance (calculated as the great circle distance between the start and end of migration), wind support, crosswinds, and vertical velocity (a proxy for uplift strength). Random effects include a random intercept for individual ID and a random slope (Age|ID) to account for repeated measures on the same individual over time. Model estimates are based on 120 spring migration events from 37 unique individuals.

**Coefficient**	**Estimate**	**95% CI**	**Standard Error**	**t value**
Intercept	172.69	118.17 – 216.06	25.02	6.90
Age	-9.63	-12.36 – -7.14	1.31	-7.35
Migration distance	-26.86	-41.20 – -9.56	8.00	-3.36
Wind support	-0.48	-2.01 – 0.83	0.71	-0.67
Vertical velocity	-9.01	-38.06 – 20.12	15.62	-0.58
Crosswinds	-0.87	-3.40 – 1.49	1.20	-0.72

Table S7. Statistical summary of the fixed effects of a linear mixed effects model examining the effect of age on the end of spring migration while accounting for potentially confounding effects of migration distance (calculated as the great circle distance between the start and end of migration), wind support, crosswinds, and vertical velocity (a proxy for uplift strength). Random effects include a random intercept for individual ID and a random slope (Age|ID) to account for repeated measures on the same individual over time. Model estimates are based on 120 spring migration events from 37 unique individuals.

**Coefficient**	**Estimate**	**95% CI**	**Standard Error**	**t value**
Intercept	99.42	39.25 – 162.04	30.55	3.25
Age	-17.04	-20.06 – -13.35	1.70	-10.03
Migration distance	10.16	-9.06 – 29.62	9.77	1.04
Wind support	-0.5-	-2.47 – 1.40	0.93	-0.54
Vertical velocity	-21.62	-63.38 – 18.41	20.27	-1.07
Crosswinds	-2.04	-5.03 – 1.22	1.57	-1.30

Table S8. Statistical summary of the fixed effects of a linear mixed effects model examining the effect of age on the difference between wind support experienced during flight and non-flight periods of migration. Random effects include a random intercept for individual ID and a random slope (Age|ID) to account for repeated measures on the same individual over time. Model estimates are based on 262 migration events (spring and fall migrations) for 40 individuals.

**Coefficient**	**Estimate**	**95% CI**	**Standard Error**	**t value**
Intercept	-0.034	-0.041 – 0.33	0.19	-0.18
Age	0.095	-0.10 – 0.29	0.095	1.00
Season	0.33	-0.15 – 0.86	0.27	1.22
Age:Season (Spring)	0.37	0.16 – 0.58	0.11	3.31

Table S9. Statistical summary of the fixed effects of a linear mixed effects model examining the effect of age on the difference between crosswinds experienced during flight and non-flight periods of migration. Random effects include a random intercept for individual ID and a random slope (Age|ID) to account for repeated measures on the same individual over time. Model estimates are based on 262 migration events (spring and fall migrations) for 40 individuals.

**Coefficient**	**Estimate**	**95% CI**	**Standard Error**	**t value**
Intercept	-0.58	-0.83 – -0.35	0.12	-4.72
Age	-0.014	-0.10 – 0.080	0.049	-0.29
Season	-0.23	-0.59 – 0.014	0.18	-1.26
Age:Season (Spring)	-0.14	-0.29 – 0.0006	0.074	-1.88

Table S10. Statistical summary of the fixed effects of a linear mixed effects model examining the effect of age on the difference between uplift strength (measured as vertical velocity) experienced during flight and non-flight periods of migration. Random effects include a random intercept for individual ID and a random slope (Age|ID) to account for repeated measures on the same individual over time. Model estimates are based on 262 migration events (spring and fall migrations) for 40 individuals.

**Coefficient**	**Estimate**	**95% CI**	**Standard Error**	**t value**
Intercept	-0.020	-0.045 – 0.0048	0.014	-1.48
Age	0.0014	-0.011 – 0.013	0.0054	0.26
Season	-0.010	-0.046 – 0.030	0.020	-0.51
Age:Season (Spring)	0.018	0.00082 – 0.034	0.0081	2.19

Table S11 Statistical summary of the fixed effects of a linear mixed effects model examining the effect of age on cumulative Overall Dynamic Body Acceleration (ODBA) during migratory flight while accounting for potentially confounding effects of wind support, crosswinds, vertical velocity (a proxy for uplift strength), and season (Fall is the reference category). Random effects include a random intercept for individual ID and a random slope (Age|ID) to account for repeated measures on the same individual over time. Unlike the standardized estimates in Figure 2, all units are unstandardized. Model estimates are based on 244 migration events (spring and fall migrations) from 40 unique individuals.

**Coefficient**	**Estimate**	**95% CI**	**Standard Error**	**t value**
Intercept	8.20	6.63 – 9.77	0.81	10.16
Age	0.70	0.37 – 1.08	0.17	4.11
Wind support	0.38	0.11 – 0.66	0.14	2.74
Crosswinds	0.19	-0.26 – 0.65	0.23	0.83
Vertical velocity	5.07	-0.38 – 10.72	2.81	1.81
Season (Spring)	3.04	2.04 – 4.04	0.51	5.98

Table S12. Statistical summary of the fixed effects of a linear mixed effects model examining the effect of age on route fidelity while accounting for potentially confounding effects of wind support, crosswinds, vertical velocity (a proxy for uplift strength), and season (Fall is the reference category). Route fidelity is calculated as the natural log of Dynamic Time Warping (DTW), which is a metric of route dissimilarity where smaller values represent higher route fidelity across migration seasons. Random effects include a random intercept for individual ID and a random slope (Age|ID) to account for repeated measures on the same individual over time. Unlike the standardized estimates shown in Figure 3, all units are unstandardized. Model estimates are based on 174 observations from 39 unique individuals.

**Coefficient**	**Estimate**	**95% CI**	**Standard Error**	**t value**
Intercept	7.69	7.41 – 7.97	0.14	56.54
Age	-0.27	-0.36 – -0.20	0.032	-8.52
Wind support	-0.033	-0.071 – 0.005	0.020	-1.68
Crosswinds	0.024	-0.041 – 0.089	0.033	0.73
Vertical velocity	0.61	-0.28 – 1.49	0.45	1.35
Season (Spring)	-0.063	-0.21 – 0.090	0.076	-0.83

Table S13. Statistical summary of the fixed effects of a linear mixed effects model examining the effect of age on route straightness while accounting for potentially confounding effects of wind support, crosswinds, vertical velocity (a proxy for uplift strength), and season (Fall is the reference category). A straightness value of one represents the most direct movement path between start and end points (i.e., the beeline). Random effects include a random intercept for individual ID and a random slope (Age|ID) to account for repeated measures on the same individual over time. Unlike the standardized estimates shown in Figure S6, all units are unstandardized. Model estimates are based on 262 migration events (spring and fall migrations) from 40 unique individuals.

**Coefficient**	**Estimate**	**95% CI**	**Standard Error**	**t value**
Intercept	0.70	0.65 – 0.74	0.023	30.92
Age	0.034	0.025 – 0.043	0.0046	7.39
Wind support	0.0025	-0.0060 – 0.011	0.0043	0.58
Crosswinds	-0.0014	-0.015 – 0.013	0.0071	-0.19
Vertical velocity	-0.11	-0.27 – 0.065	0.086	-1.21
Season (Spring)	0.018	-0.014 – 0.049	0.016	1.11

Table S14. Statistical summary of the fixed effects of a linear mixed effects model examining the effect of age on migration distance while accounting for potentially confounding effects of wind support, crosswinds, vertical velocity (a proxy for uplift strength), and season (Fall is the reference category). Random effects include a random intercept for individual ID and a random slope (Age|ID) to account for repeated measures on the same individual over time. Migration distance is calculated as the great circle distance in km between the start and end of migration. Model estimates are based on 262 migration events (spring and fall migrations) from 40 unique individuals.

**Coefficient**	**Estimate**	**95% CI**	**Standard Error**	**t value**
Intercept	3.05	2.97 – 3.13	0.042	73.21
Age	-0.0062	-0.022 – 0.0084	0.0071	-0.88
Wind support	0.0059	-0.0066 – 0.018	0.0064	0.92
Crosswinds	0.0092	-0.011 – 0.030	0.010	0.89
Vertical velocity	0.075	-0.18 – 0.33	0.13	0.57
Season (Spring)	0.013	-0.031 – 0.056	0.023	0.56

Table S15. The name and version of R packages used in analysis.

**Package name**	**Version**
Packages used for statistical analysis and figure creation:	
ggforce	0.4.1
ggplot2	3.4.1
lme4	1.1-31
lubridate	1.9.2
sjstats	0.18.2
sinaplot	1.1.0
vioplot	0.4.0
Packages used for spatial analysis:	
BBMM	3.0
raster	3.6-14
rgdal	1.6-4
rgeos	0.6-1
SimilarityMeasures	1.4
sp	1.6-0
trajr	1.5.0
